# Supplementary material for: Wood-based superblack
Source: Nat Commun. 2023 Dec 5;14:7875. doi: 10.1038/s41467-023-43594-4 (PMC10697973; doi:10.1038/s41467-023-43594-4)
Supplement: Supplementary file 1 — Supplementary Information [file 41467_2023_43594_MOESM1_ESM.pdf]

Supplementary Information for

Wood-based Superblack

Bin Zhao[1], Xuetong Shi[2], Sergei Khakalo[3,4], Yang Meng[5], Arttu Miettinen[6], Tuomas Turpeinen[7], Shuyi Mi[8], Zhipei Sun[8,9], Alexey Khakalo[10], Orlando J. Rojas[1,2,*], Bruno D. Mattos[1,*]

[1]Department of Bioproducts and Biosystems, School of Chemical Engineering, Aalto University; Espoo, FI-02150, Finland
[2]Bioproduct Institute, Department of Chemical & Biological Engineering, Department of Chemistry and Department of Wood Science, The University of British Columbia; Vancouver, BC V6T 1Z3, Canada
[3]Department of Civil Engineering, School of Engineering, Aalto University; Espoo, FI-02150, Finland
[4]Integrated Computational Materials Engineering, VTT Technical Research Centre of Finland Ltd.; Espoo, FI-02044, Finland
[5]Faculty of Chemical Engineering, Kunming University of Science and Technology; Kunming, 650500, PR China
[6]Department of Physics, University of Jyvaskyla; Jyväskylä, FI-40014, Finland
[7]Fiber web processes, VTT Technical Research Centre of Finland Ltd.; Jyväskylä, FI-40400, Finland
[8]Department of Electronics and Nanoengineering, Aalto University; Espoo, FI-02150, Finland.
[9]QTF Centre of Excellence, Department of Applied Physics, Aalto University; Espoo, FI-02150, Finland
[10]Functional Cellulose, VTT Technical Research Centre of Finland Ltd.; Espoo, FI-02044, Finland

*Authors for correspondence: Bruno D. Mattos, E-mail: bruno.mattos@aalto.fi
Orlando J. Rojas, E-mail: orlando.rojas@ubc.ca

This PDF includes:

Supplementary Sections 1 to 3

Supplementary Figures 1 to 30

Supplementary Table 1

Supplementary References

Supplementary Section 1. Finite element modeling of the optical properties of cellular structures.

Simulation setup.

In the framework of continuum description of condensed matter, finite element (FE) software COMSOL Multiphysics® 5.6.0.401 (Electromagnetic Waves, Frequency Domain in Wave Optics Module[1]) is used for modeling the optical properties of cellular structures. In total, 13 cases are studied. The two-dimensional simulation setup for Case 1 is shown in Supplementary Figure 1a. In Case 1, taken as a reference case, we analyze the optical properties of a flat surface. It is modeled by considering a two-dimensional unit cell with a width of 1 μm. The model consists of three parts. The first part (highlighted in blue) represents a graphite material, the second part (grey color) is used for air, and the third part (marked as green) defines perfectly matched layers (PMLs). For air, the refractive index is real and takes the value 1, while graphite has wavelength-dependent real and imaginary parts of refractive index[2]. Floquet periodic boundary conditions (PBCs) are applied on the left and right sides of the computational domain. The interior boundary with a Port BC with active domain-backed slit condition (yellow line in Supplementary Figure 1a) is placed 2 μm above the air-graphite interphase and used to launch a plane wave incident at 0 degrees with port input power of 1 W/m and wavelengths in a range from 350 nm to 800 nm. PMLs are used to absorb all reflected light (top layer) and transmitted light (bottom layer). Two additional interior boundaries (red lines in Supplementary Figure 1a) are introduced to monitor the total reflected and transmitted light by integrating the power flux normalized with respect to the incident power. The interior boundaries are placed in front of (0.1 μm away from) the PML domains. The absorbance within the graphite is computed by integrating the losses within the graphite domain. Quadratic discretization is used for electric field. Free triangular and mapped mesh options are selected with minimum and maximum element size, respectively, of 0.9 nm and 35 nm. To assure accurate integration, a boundary layer mesh option is used for the interior boundaries with the following properties: number of boundary layers is 4, boundary layer stretching factor is 2, and thickness of first layer is 1 nm. More details can be found in a COMSOL Blog on modelling the optical properties of rough surfaces[3]. Distribution of the electric field norm for a reference Case 1 is shown in Supplementary Figure 1b.

In Cases 2-3 and 6-15, we analyzed the optical properties of cellular structures. The structures are modelled as a regular 2D array of straight rectangular (or trapezoidal) pillars with a skew bottom part. Domain occupied by a graphite material is highlighted in blue, while grey color is used for domain occupied by air. Simulation setup is similar to Case 1 except of the unit cell size and composition.

To investigate the light interactions with wood anatomical elements, 2D vertical cylinder array of straight rectangular pillars was employed in the simulation (Supplementary Figure 2a). Light reflectance on 2D cylinder array with varied fiber length (spanning 20-200 μm), cell wall thickness (spanning 0.7-6 μm), lumen width (spanning 7.5-60 μm), and cell end tilt angle (spanning 0-60 °) are studied in Cases 2-3, 6-12 and 14. The dimension of the anatomical elements used in the simulation was extracted from the microCT scans (Supplementary Figure 7) and SEM images (Supplementary Figures 15-20) of balsa wood. Case 2 examines the total light reflectance of cylinder array as a function of cell end tilt angle. Cases 1, 2, 3, 8 and 9 show the effect of cell wall thickness on the light reflectance of cylinder array. Cases 2, 10, 11 and 12 examine the carbonized wood-light interactions in cylinder array with varied fiber length. Cases 2, 6, 7 and 14 study the effect of lumen width on the light reflectance. To verify the contribution of new subwavelength structures to light absorption, 2D cylinder array of trapezoidal pillars is employed in both Cases 13 and 15 (Supplementary Figures 5-6). The dimension of the short base and the long base of the trapezoidal pillars is 0.2 μm and 1.5 μm, respectively, which is used in both Cases 13 and 15. The altitude of trapezoidal pillars is set at 30 μm and 100 μm, which corresponds to the height of bandsaw-like microarrays in NW carbon (in Case 13) and carbon microfiber arrays in superblack wood (Case 15), respectively. To create the interfiber porosity of tens of microns, one blackbody-like cell unit is installed adjacent to the regular cylinder array (Supplementary Figure 6b).

Case studies and results.

For Cases 2 and 3, the pillars have height 120 μm and are placed in space with a step of 15 μm. The pillars have width 1.5 μm (Case 2) and 6 μm (Case 3). The corresponding unit cells (shown in Supplementary Figure 2a and 2b) are selected such that they include halves of the neighbouring pillars. The bottom part (in its horizontal position) is placed 100 μm below the pillars top surface (H=100 μm). The bottom slope is introduced by rotating the bottom part

around a middle point at the upper surface of the bottom part. The rotation angle is taken in a range from 0° to 60° with 5° increment. For fixed height and spacing between the pillars, Cases 2 and 3 study the influence of the pillar width (representing cell wall thickness in wood) and slope of a bottom part (representing cell end tilt angle in wood) on the reflective properties. For Case 2, with the selected bottom slopes 0°, 15°, 30°, 45°, and 60°, distribution of the electric field norm is presented in Supplementary Figure 2c for wavelength λ=350 nm and in Supplementary Figure 2d for wavelength λ=800 nm. Reflectance as a function of wavelength is plotted in Supplementary Figure 2e for Cases 2 (including Case 1 and Case 3, φ=30 °).

For Cases 6-9, the pillars have height 120 μm and are placed in space with a step of 7.5 μm (Case 6, H=100 μm, W=7.5 μm), 30 μm (Case 7, H=100 μm, W=30 μm), and 15 μm (Cases 8 and 9, H=100 μm, W=15 μm). The pillars have width 1.5 μm (Cases 6 and 7), 1 μm (Case 8), and 0.7 μm (Case 9). The corresponding unit cells are shown in Supplementary Figure 3a-3d. The bottom part (in its horizontal position) is placed 100 μm below the pillars top surface (H=100 μm) and then rotated by 30° around a middle point at the upper surface of the bottom part. For fixed height of the pillars and slope of the bottom part, Cases 6-9 study the influence of the pillar width (representing cell wall thickness in wood) and spacing between the pillars (representing lumen width in wood) on the reflective properties. Distribution of the electric field norm for wavelengths λ=350 nm and λ=800 nm is presented in Supplementary Figure 3e for Case 6, in Supplementary Figure 3f for Case 7, in Supplementary Figure 3g for Case 8, and in Supplementary Figure 3h for Case 9. Reflectance as a function of wavelength is plotted in Supplementary Figure 3i (including results for Case 2, φ=30° and Cases 10-12).

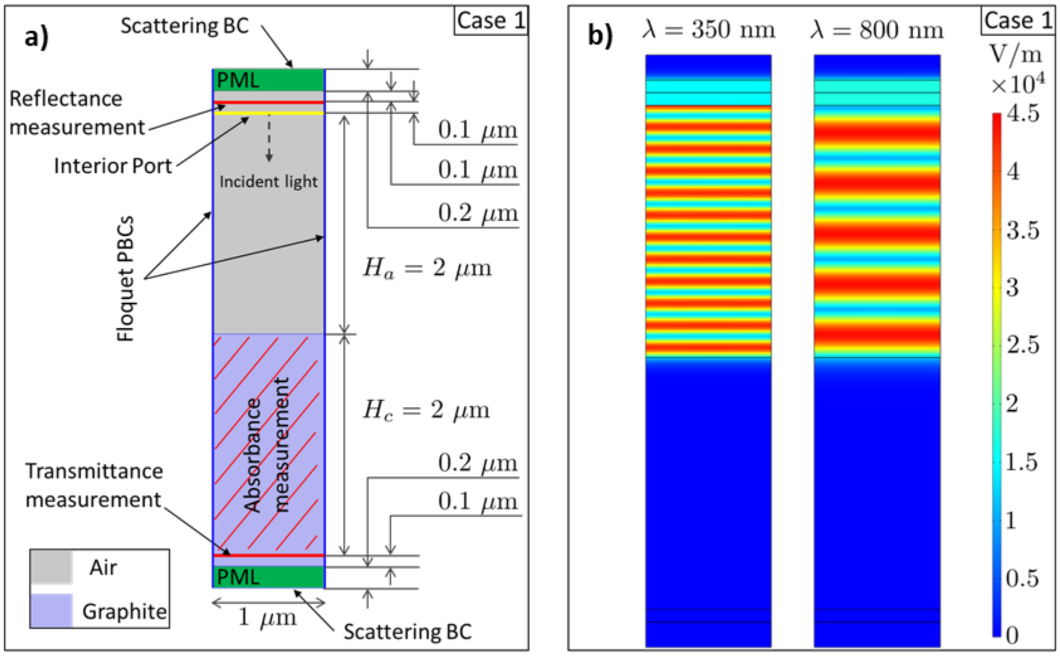

Supplementary Figure 1. FEM simulation setup for Case 1. a Computational model. **b** Distribution of the electric field norm for wavelengths λ=350 nm and λ=800 nm.

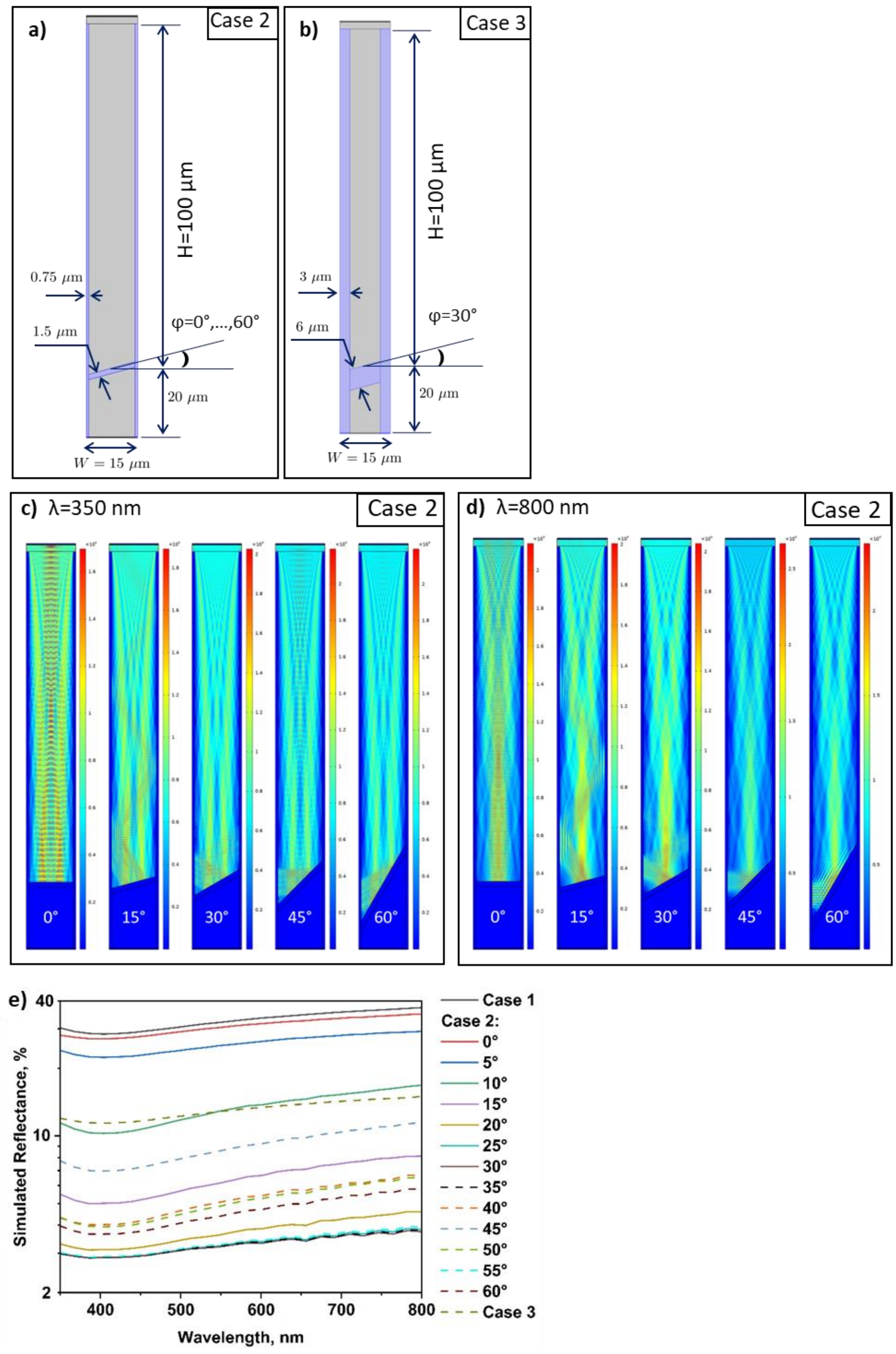

Supplementary Figure 2. FEM simulation setup for Cases 2 and 3. Computational model for **a** Case 2 and for **b** Case 3. Distribution of the electric field norm for Case 2 at wavelengths **c** λ=350 nm and **d** λ=800 nm. **e** Reflectance as a function of wavelength for Cases 1-2, and Case 3, φ=30 °.

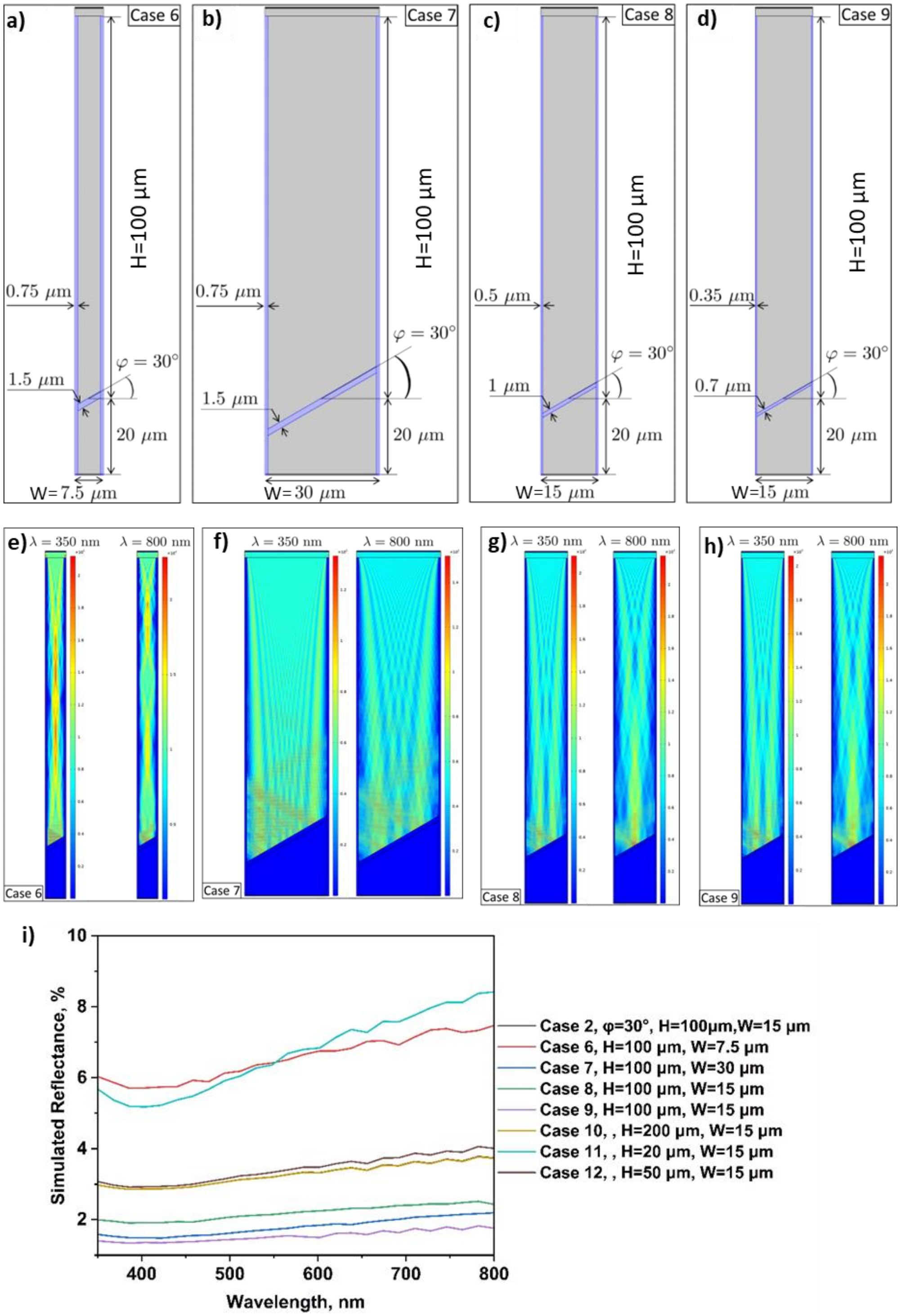

Supplementary Figure 3. FEM simulation setup for Cases 6, 7, 8, and 9. Computational model for **a** Case 6, for **b** Case 7, for **c** Case 8, and for **d** Case 9. Distribution of the electric field norm for wavelengths λ=350 nm and λ=800 nm for **e** Case 6, for **f** Case 7, for **g** Case 8, and for **h** Case 9. **i** Reflectance as a function of wavelength for Cases 6-12 (including Case 2, φ=30°).

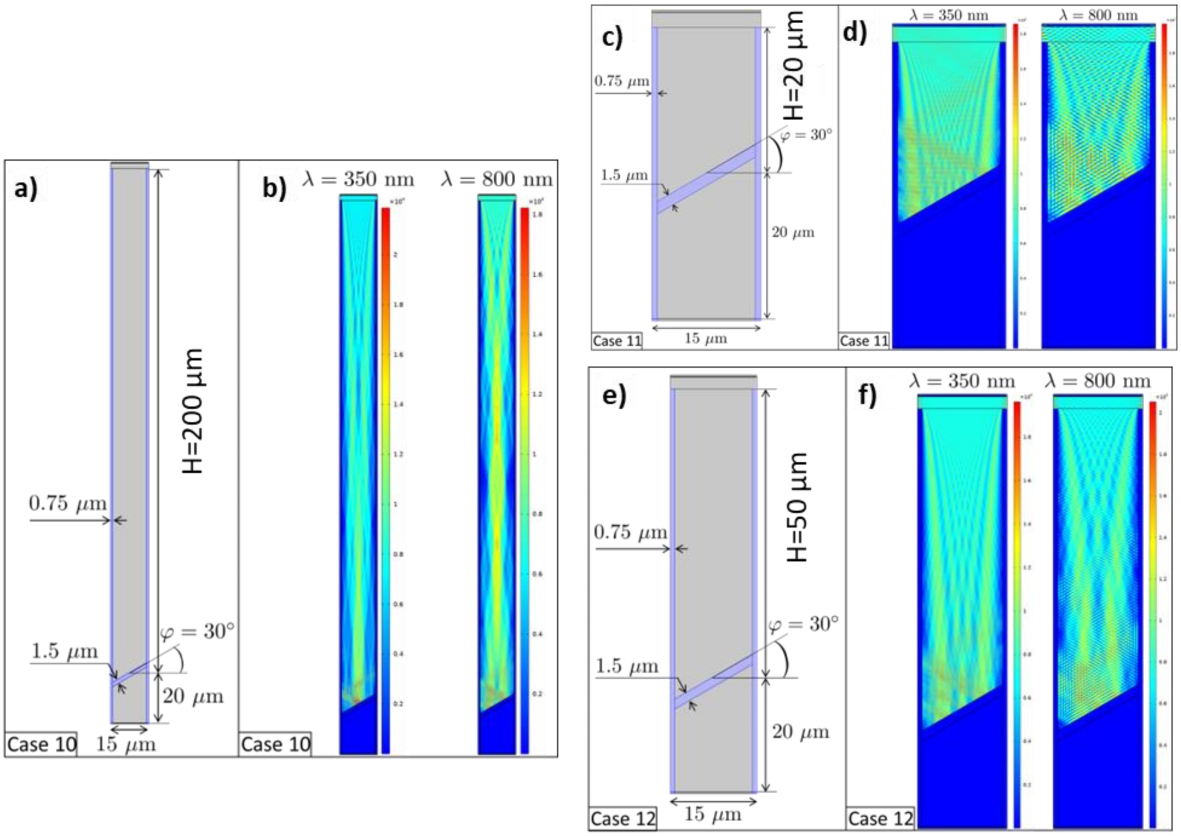

Supplementary Figure 4. FEM simulation setup for Cases 10, 11, and 12. Computational model for **a-b** Case 10, for **c-d** Case 11, and for **e-f** Case 12. Distribution of the electric field norm for wavelengths λ=350 nm and λ=800 nm for **b** Case 10, for **d**) Case 11, and for **f** Case 12.

For Cases 10-12, the pillars have height 220 μm, 40 μm, and 70 μm, respectively, and are placed in space with a step of 15 μm. The pillars have width 1.5 μm. The corresponding unit cells are shown in Supplementary Figure 4a, 4c, and 4e. The bottom part (in its horizontal position) is placed 200 μm (for Case 10, H=200 μm, W=15 μm), 20 μm (for Case 11, H=20 μm, W=15 μm), and 50 μm (for Case 12, H=50 μm, W=15 μm) below the pillars top surface and then rotated by 30° around a middle point at the upper surface of the bottom part. For fixed slope of the bottom part, width and spacing between the pillars, Cases 10-12 study the influence of the pillar height (representing fiber length in wood) on the reflective properties. Distribution of the electric field norm for wavelengths λ=350 nm and λ=800 nm is presented in Supplementary Figure 4b for Case 10, in Supplementary Figure 4d for Case 11, and in Supplementary Figure 4f for Case 12.

Reflectance as a function of wavelength is plotted in Supplementary Figure 3i (including results for Case 2, φ=30° and Cases 6-9).

For Case 13, the pillars have shape of right trapezoid with top and bottom sides of 0.2 μm and 1.5 μm, respectively. The corresponding unit cells are schematically shown in Supplementary Figure 5a. The height of the trapezoidal pillars is 30 μm between the pillars top surface and the straight rectangular pillars. The total pillars height takes values H=120 μm below the pillars top surface. Width between the pillars takes W=40 μm for Case 13. Distribution of the electric field norm for wavelengths λ=350 nm and λ=800 nm is presented in Supplementary Figure 5c for Case 13. For Case 14, the pillars are straight rectangles with width 1.5 μm. The pillars have height H+20 μm (representing fiber length in wood) and distance between the pillars (representing lumen width in wood) is W. The corresponding unit cells are schematically shown in Supplementary Figure 5b. The bottom part (in its horizontal position) is placed below the pillars top surface by distance H and then rotated by 30° around a middle point at the upper surface of the bottom part. Distance H takes values H=100 μm and H=200 μm for Case 14. Width between the pillars takes W=40 μm and W=60 μm. Distribution of the electric field norm for wavelengths λ=350 nm and λ=800 nm is presented in Supplementary Figure 5d for Case 14 (with H=200 μm and W=60 μm). Reflectance as a function of wavelength is plotted in Supplementary Figure 5e for Case 13 (including Case 14, H=100 μm and W=40 μm) and in Supplementary Figure 5f for Case 14 (including Case 2, φ=30°, Cases 6 and 7).

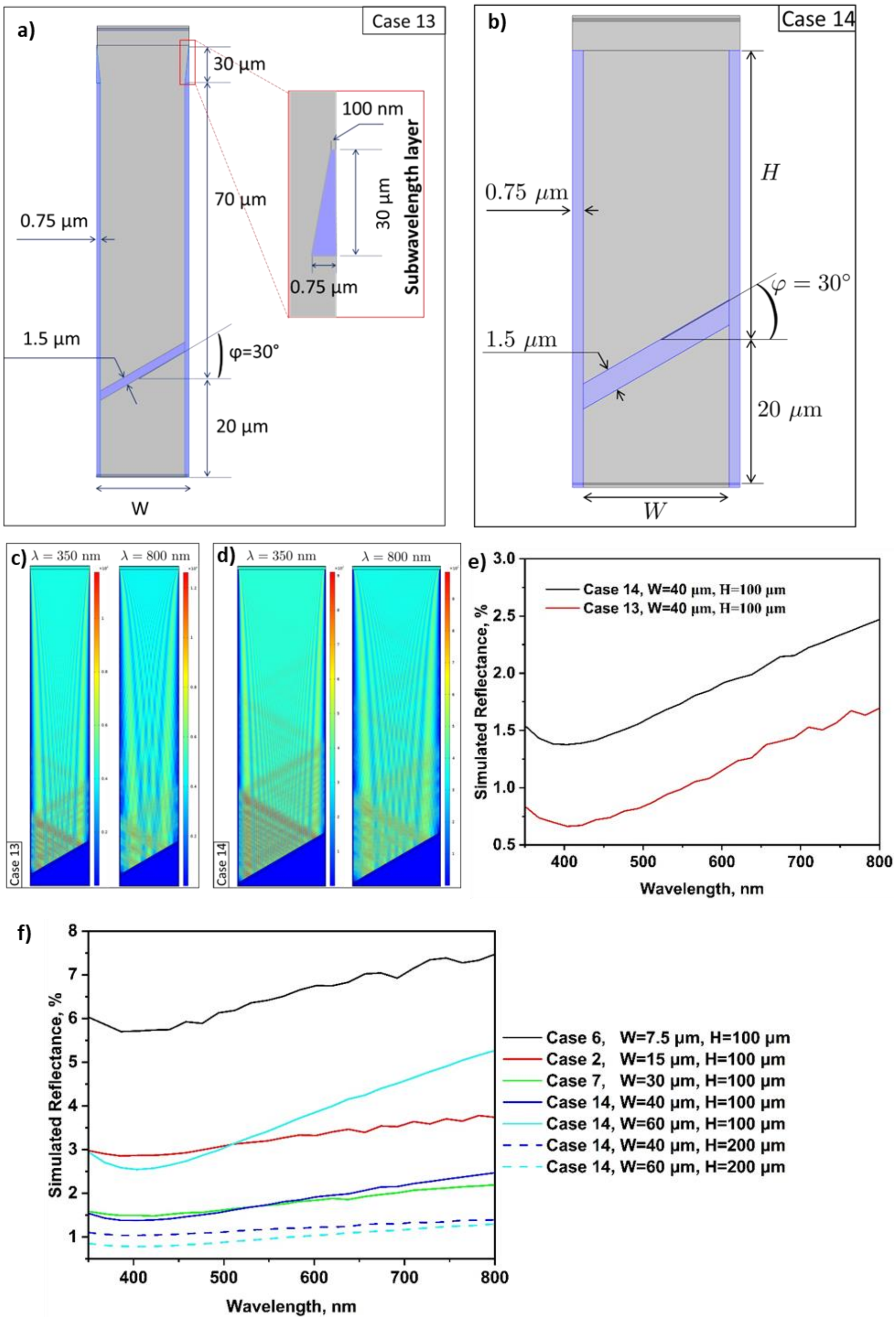

Supplementary Figure 5. FEM simulation setup for Cases 13 and 14. a Computational model for Case 13. Total pillar height takes values H=120 µm while the height for the trapezoidal parts take value 30 µm. Distance between the pillars at bottom part takes values W=40 µm. **b** Computational model for Case 14. Pillar height takes values H=100 µm and H=200 µm. Distance between the pillars takes values W=40 µm and W=60 µm. Distribution of the electric field norm for wavelengths λ=350 nm and λ=800 nm for **c** Case 13, for **d** Case 14 with H=200 µm and W=60 µm. Reflectance as a function of wavelength for **e** Case 13 and for **f** Case 14 (including results from Cases 2, 6 and 7, H=100 µm).

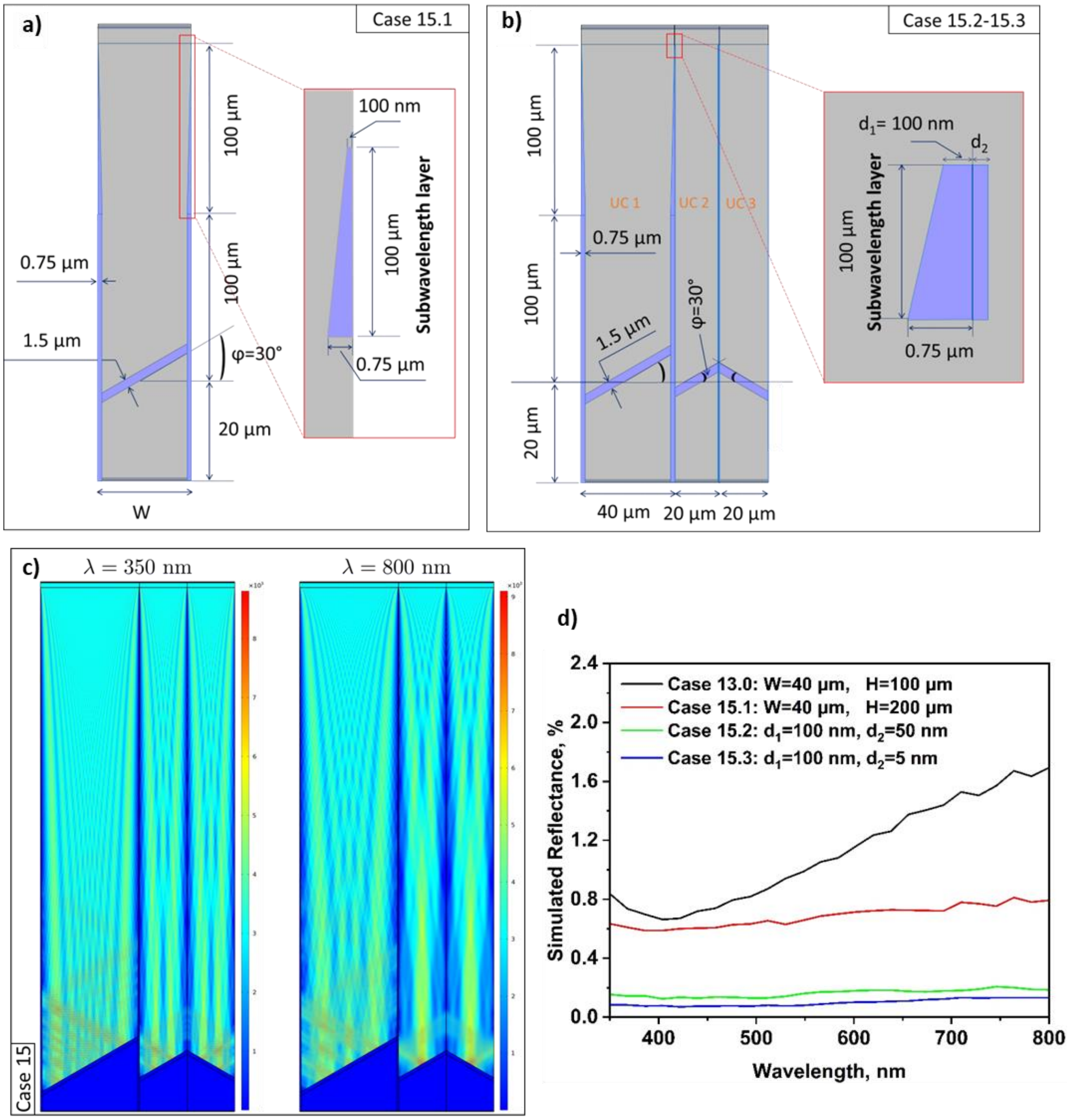

Supplementary Figure 6. FEM simulation setup for Case 15. a-b Computational model details. **c** Distribution of the electric field norm for wavelengths λ=350 nm and λ=800 nm for Case 15.3: d_1=100 nm and d_2=5 nm. d Reflectance as a function of wavelength.

For Case 15.1, the cylinder array has trapezoidal pillars with top and bottom sides of 0.2 μm and 1.5 μm, respectively. The height of the trapezoidal pillars between the topmost surface and the straight rectangular pillars is 100 μm as schematically shown in Supplementary Figure 6a. The total pillar height takes values H=220 μm below the pillars top surface. The bottom part (in its horizontal position) is placed 200 μm below the pillars top surface and then rotated by 30° around a middle point at the upper surface of the bottom part. Width between the pillars takes

W=40 μm. For Cases 15.2 and 15.3, the computational model combines three-unit cells as schematically shown in Supplementary Figure 6b. Unit cell UC 1 represents a cylinder array of the trapezoidal pillars with the bottom side of 1.5 μm. The top side has width 2 d_1 where d_1 takes value d_1=100 nm and the height of the trapezoidal pillars is 100 μm, which corresponds to unit cell in Case 15.1. Unit cells UC 2 and UC 3 represent a regular array of straight rectangular pillars with width 2 d_2 where d_2 takes values d_2=50 nm and d_2=5 nm for Cases 15.2 and 15.3, respectively. The pillars have height 220 μm and distance between the pillars is 40 μm for UC 1 and 20 μm for both UC 2 and UC 3. The bottom part (in its horizontal position) is placed 200 μm below the pillars top surface and then rotated by 30° (in UC 1 and UC 2) and by -30° (in UC 3) around a middle point at the upper surface of the bottom part. Distribution of the electric field norm for wavelengths λ=350 nm and λ=800 nm is presented in Supplementary Figure 6c for Case 15.3: d_1=100 nm and d_2=5 nm. Reflectance as a function of wavelength is plotted in Supplementary Figure 6d (including results for Case 13).

The studies demonstrate that the light reflectance decreases when the cell end tilts at larger angles (up to 30º) (Fig. 2f-2g and Case 2 shown in Supplementary Figure 2e). The studies conclude that light reflectance positively correlates with cell wall thickness (Fig. 2h and Cases 1, 2, 3, 8, and 9 shown in Supplementary Figure 2e and 3i). The studies demonstrate that larger lumen width also favors the overall light trapping and lowers the light reflectance (Fig. 2i and Cases 2, 6, and 7 shown in Supplementary Figure 5f). It is also noted that nanolevel sharp edges showing low backscattering[4, 5] and large openings of tens microns[5, 6, 7] are also desirable for reaching supreme-black level. The studies indicate that a fiber length of 50 μm is enough to inhibit light escaping from the cylindrical arrays with a lumen width of 15 μm (Cases 2, 10, 11, and 12 shown in Supplementary Figure 3i and 4). However, with a larger lumen, *e.g.*, width of 40 μm, light confinement fails in cylindrical arrays with a fiber length of 100 μm (Case 14 shown in Supplementary Figure 5). With enlarged fiber length of 200 μm, cylindrical arrays with lumens width of 40 μm and 60 μm display efficient light trapping property. In balsa wood, the fiber length could be up to 800 μm considering the maximum fiber length[8, 9], which favors effective multiple internal light reflections. Microcavity of high aspect ratios is essential to reach the blackness level for supreme-black[4, 5, 7] and nickel-phosphorus (Ni-P) superblack[10], which requires heavy etching steps in the fabrication process. As-grown VANTA with inter CNT distance of tens of nm do not favor multiple internal light reflections[11, 12, 13]. Plasma treatment of

as-grown CNT forest induces aggregate of CNT and creating microcavity between CNT clusters, which facilitates multiple internal light reflections and reduce the light reflectance from 450 to 94 ppm[14, 15]. Effective multiple internal light reflections plays a crucial role but requires heavy etching steps[5, 16] and plasma treatment[15] in many superblack materials, which is enabled by the intrinsic cellular structure in superblack wood.

Combined with Case 14, the studies in Case 13 verify the contribution of 30 μm high trapezoidal pillars, corresponding to bandsaw-like microarrays in NW carbon, to light reflectance as shown in Fig. 3i and Supplementary Figure 5e. Combined with Case 13, the studies in Cases 15 verify the contribution of 100 μm high trapezoidal pillars, corresponding to microfiber arrays with interfiber porosity in superblack wood, to light reflectance as shown in Fig. 4j and Supplementary Figure 6d.

The simulation results have verified the contribution of wood anatomical elements to the light reflectance. In addition, simulation also demonstrates the contribution of the new subwavelength structures to the light absorption in both NW carbon and superblack wood. However, wood structure is heterogeneous in nature and their dimension and geometry vary with their ages and density. It is hard to find out of a set of geometric parameters that perfectly represents wood. For instance, fiber length is essential factor determining the light absorption property in cylinder arrays. However, it is hard to estimate the average fiber length for wood blocks which may vary from zero to the maximum length of 800 μm due to the heterogenous distribution of fibers along the grain. In addition, ray cells having fiber length of <30 μm, which usually shows failed multiple internal light reflections, is also not considered in the simulation setups. The idealized simulation setups are much simpler than the complex and heterogeneous subwavelength structures in superblack wood. Therefore, the minimum reflectance value of 0.08 % in the simulation is much lower than the minimum light reflectance of 0.4 % for superblack wood in the experiments.

Supplementary Section 2. Supplementary Figures and related supplementary discussions.

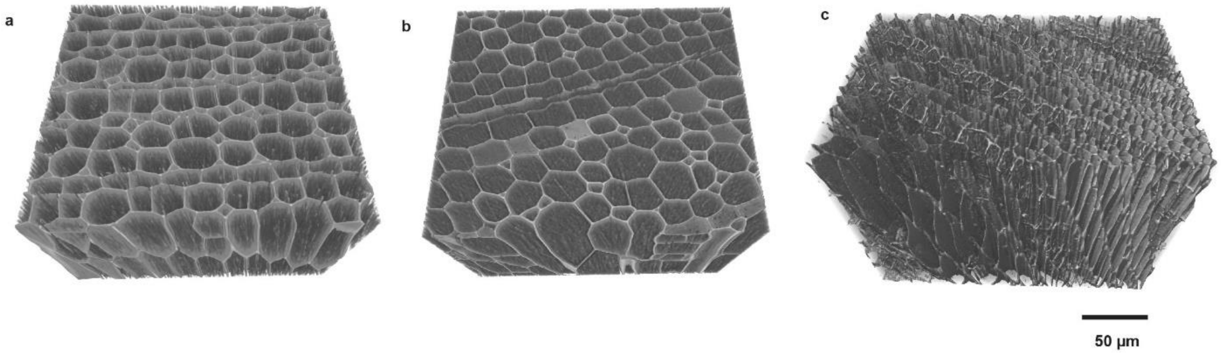

Supplementary Figure 7 Microtomography images of: **a** Wood (W), **b** Delignified wood (DW), **c** Delignified wood carbonized at 1500 ºC (cDW).

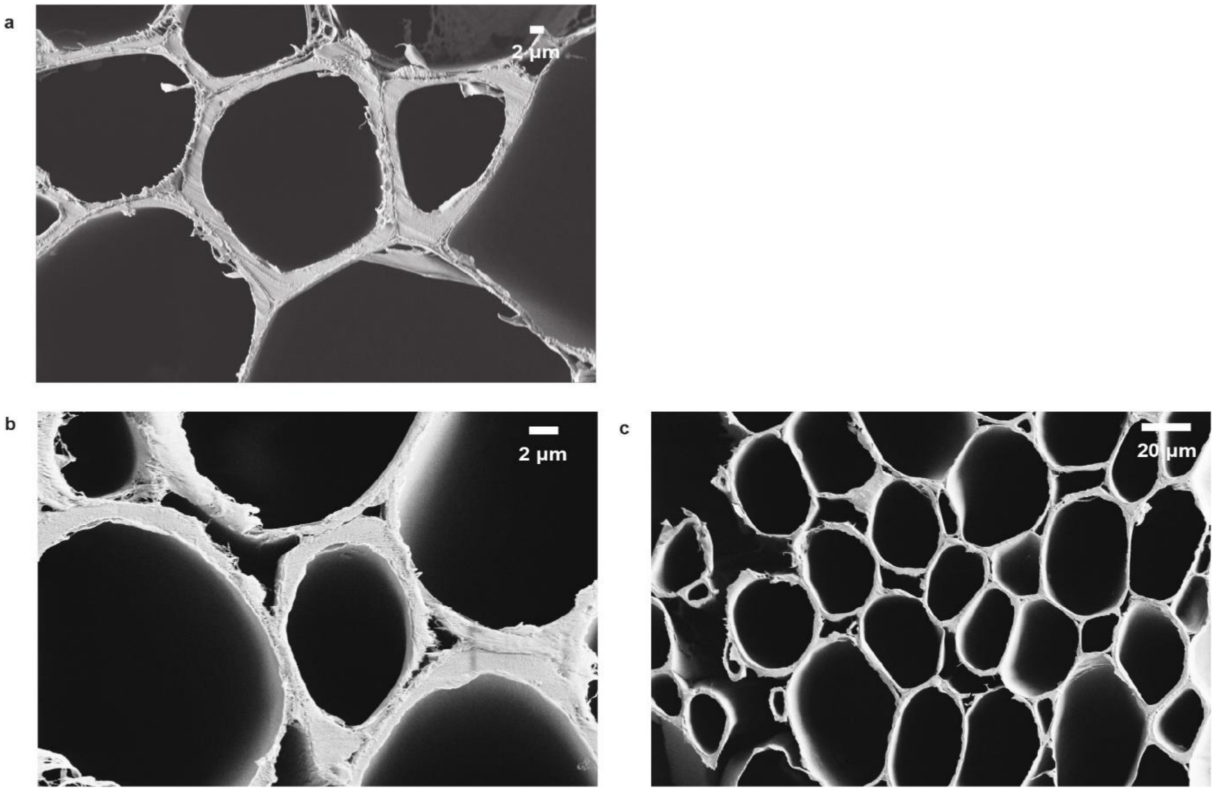

Supplementary Figure 8. SEM image showing the morphology of cell walls in **a** wood (W) and delignified wood (DW) that are frozen at -20 °C **b** and by liquid N_2 (**c**) before lyophilization.

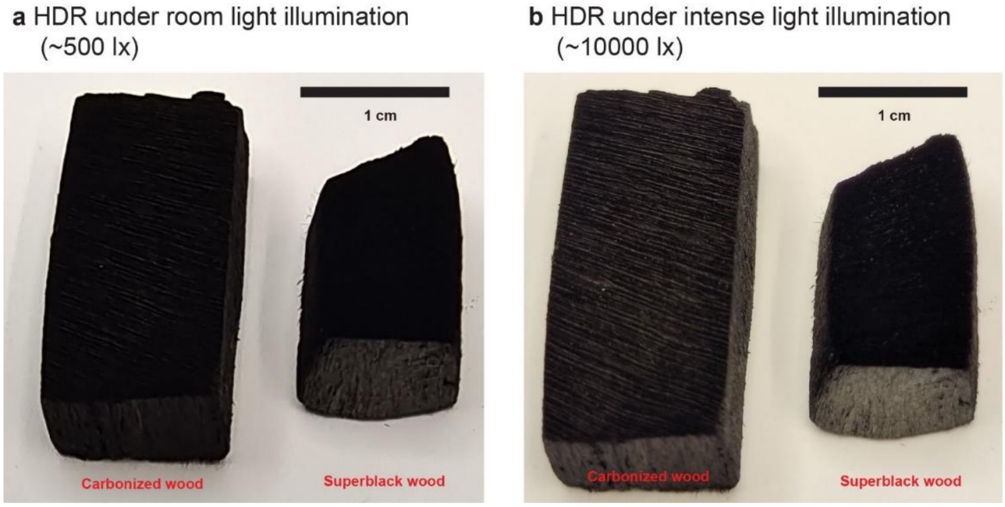

Supplementary Figure 9. Carbonized wood (cW), and Superblack wood photographed under **a** room light illumination (~500 lx) and **b** intense light illumination (~10000 lx) in high dynamic range (HDR).

Dynamic range is a measure of the light intensities from the highlights to the shadows. High dynamic range (HDR) whose synonyms are wide dynamic range is a dynamic range higher than standard dynamic range (SDR). In HDR mode, more surface features from low-reflectance samples (e.g., Superblack) can be captured, which otherwise appear pure black. Human eyes, image sensors, display devices and printed media have typical dynamic ranges of 5, 4, 3 and 2 orders of magnitude, respectively. The higher dynamic range the camera has, the closer the photos will compare to what human eyes can see. Although the light strips on cW can be observed by human eyes, the camera cannot capture them. The light stripes are hardly spotted when cW is photographed under room light illumination (~500 lx) (Supplementary Figure 9a). To capture more details, cW and Superblack wood are photographed under intense light illumination (~10000 lx) in HDR mode (Supplementary Figure 9b). The light stripes on cW become clearly visible under intense light illumination (~10000 lx). The light stripes on cW result from the carbonized ray cells in the form of flat structures with the order of tens of microns in width (Fig. 2b shown in green color and Fig. 3c). However, these light strips disappeared in Superblack wood, and cross section appear as pure black surface even under intense light illumination (~10000 lx) (Supplementary Figure 9b). In cDW, the ray cells shrunk significantly and were converted into ca. 1 µm-wide thin bands (Fig. 4d-4e, shown in green color), which explain well the disappearance of the light strips in superblack wood.

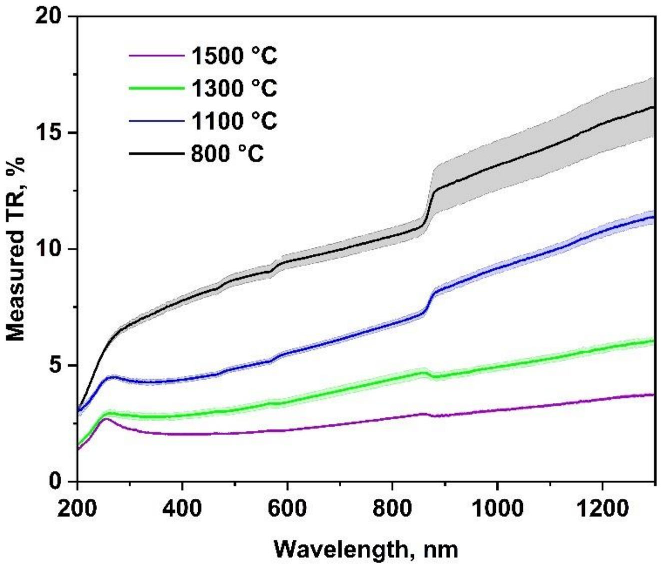

Supplementary Figure 10. Total light reflectance (TR) of radial section in wood carbon (cW) obtained at 800-1500 ºC. Shade colors represent standard deviation.

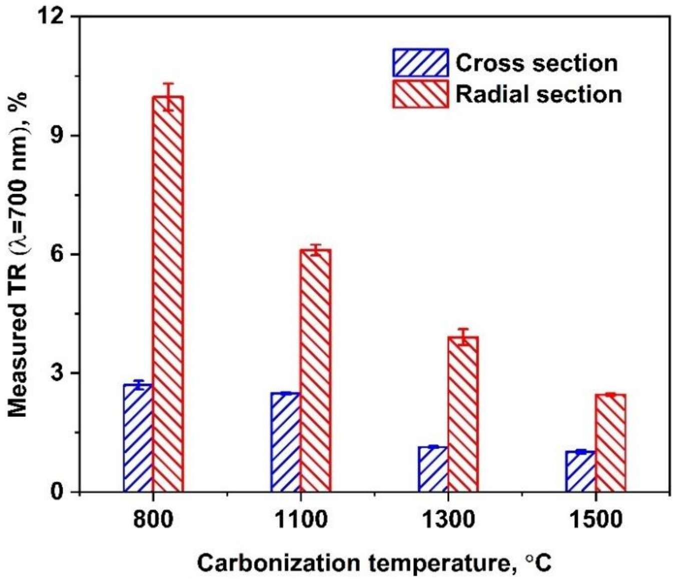

Supplementary Figure 11. Total light reflectance (TR) at 700 nm of cross section and radial section in wood carbon that were obtained at 800-1500 ºC (cW). Error bars refer to standard deviation.

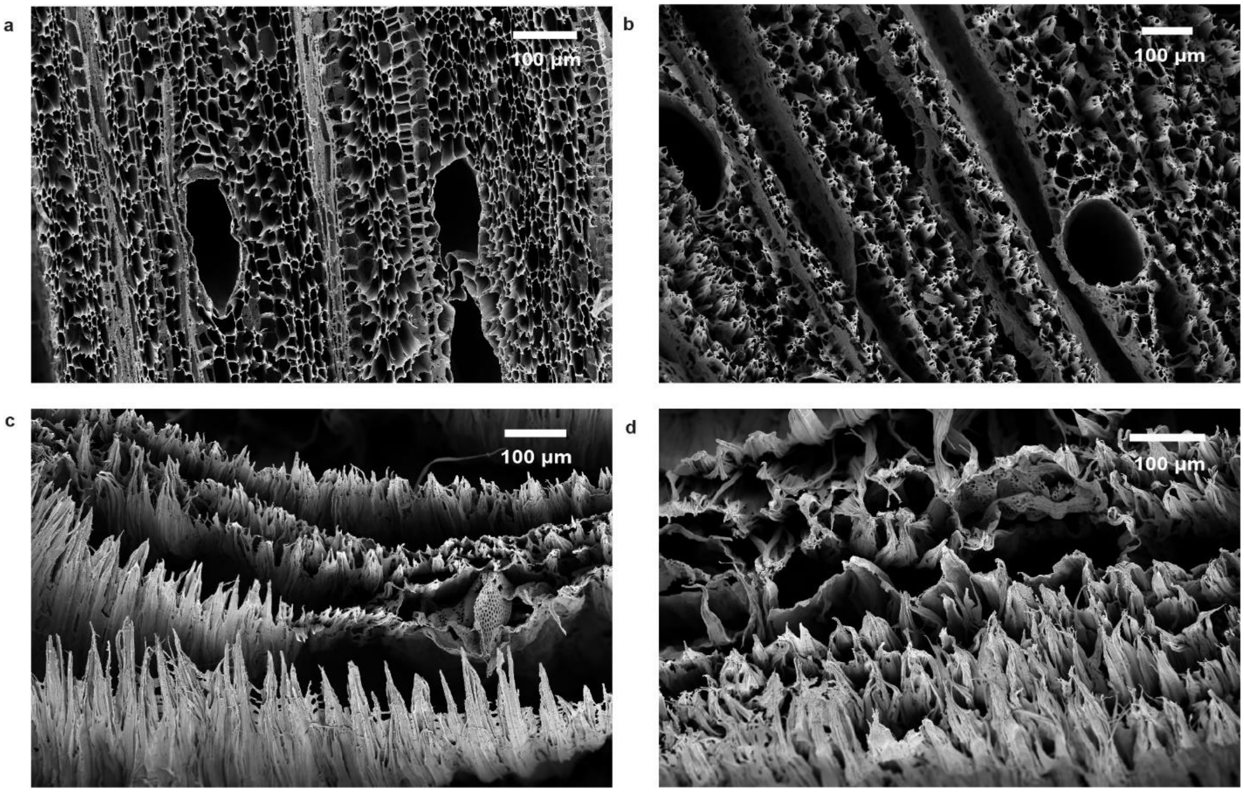

Supplementary Figure 12. SEM images showing the cross-section morphology of wood carbon (cW) that were obtained at 1500 ºC. The delignification times of **a** 0 h, **b** 1 h, **c** 2 h, and **d** 3 h.

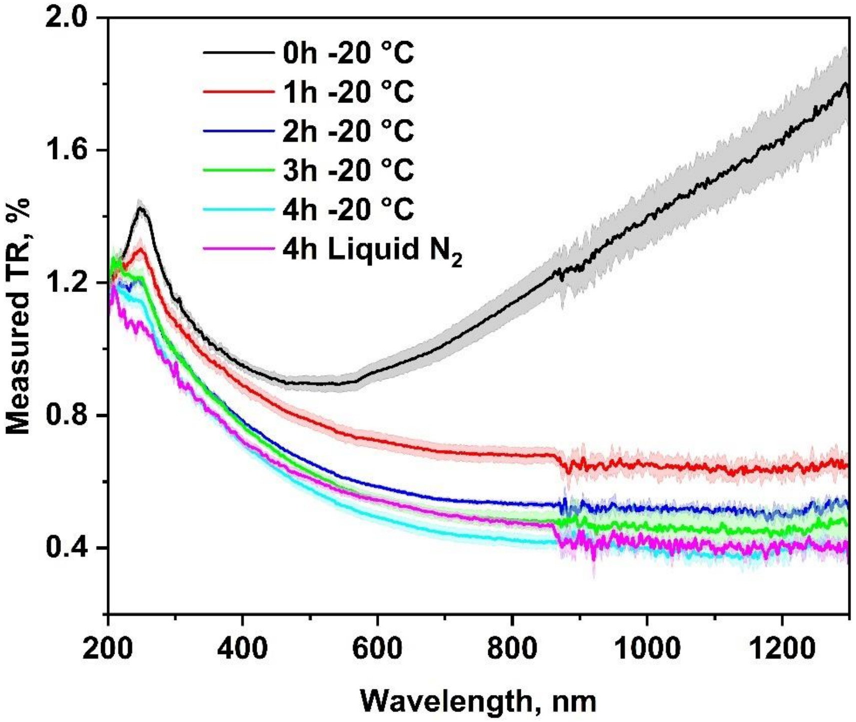

Supplementary Figure 13. Total light reflectance (TR) of cross section in cDW that were obtained at 1500 ºC. Wood was delignified with time varied from 0 to 4h. Delignified wood are frozen either at -20 °C or by liquid N_2 before lyophilization. Shade colors represent standard deviation.

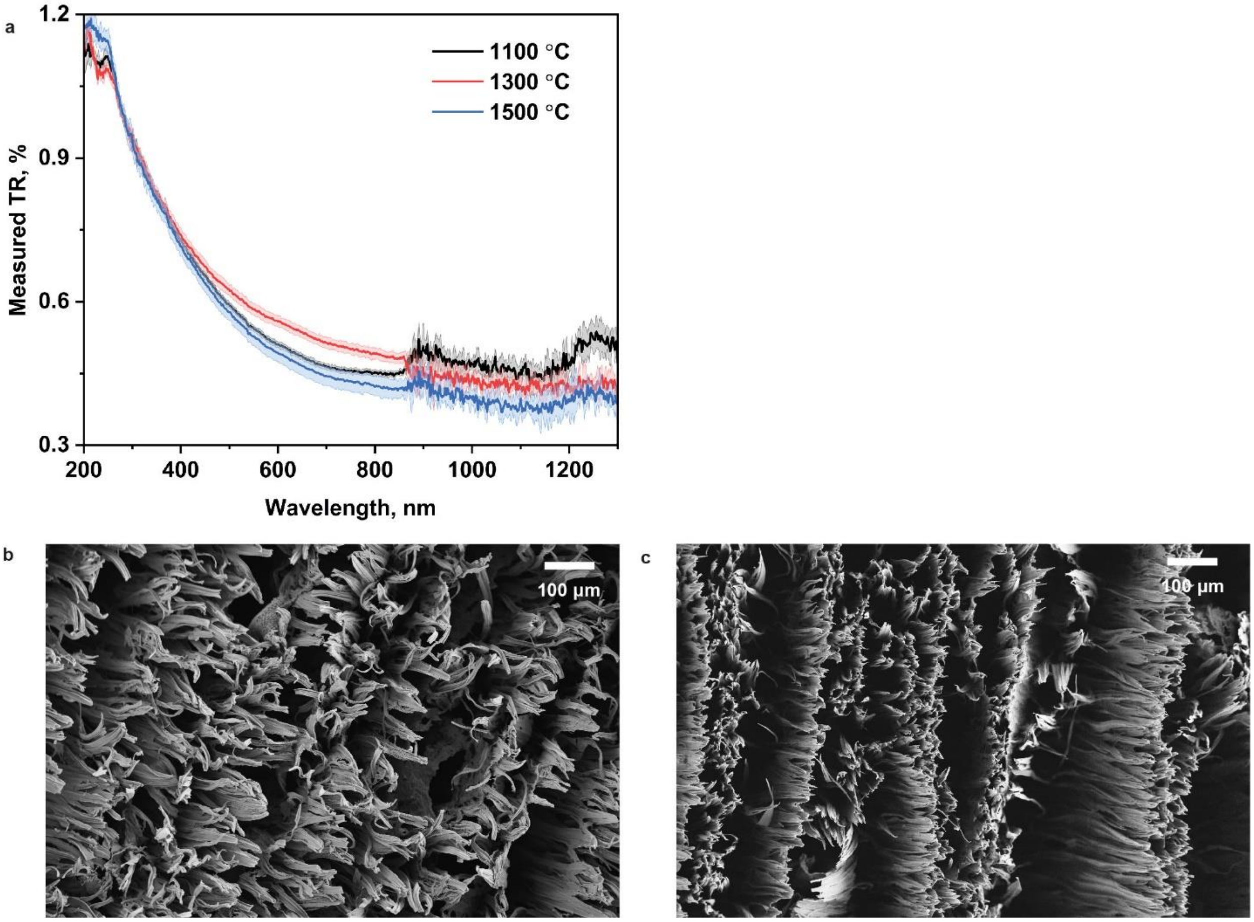

Supplementary Figure 14. a Total light reflectance (TR) of cross section in delignified wood that carbonized at 1100-1500 ºC, cDW. **b** SEM image showing curved and deformed carbon microfiber arrays in cDW obtained at 1100 ºC. **c** SEM image showing vertically aligned carbon microfiber arrays in cDW obtained at 1300 ºC. Shade colors in **a** represent standard deviation.

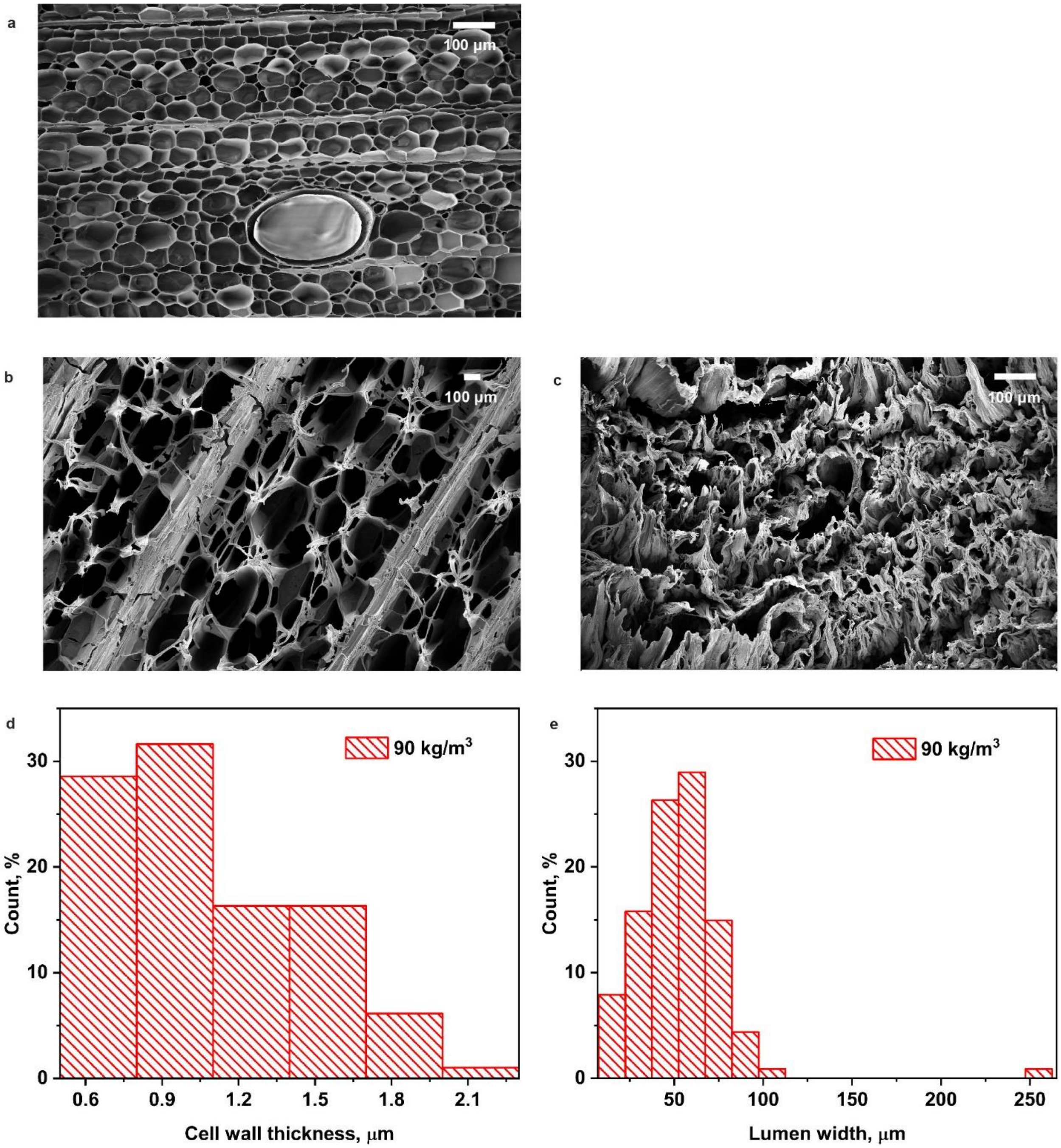

Supplementary Figure 15. Top-view SEM images showing the cross-section morphology of **a** wood (W), **b** cW and **c** cDW. Both cW and cDW were obtained at 1500 ºC. The distribution of **d** cell wall thickness and **e** lumen width in balsa wood (W) with a density of 90 kg/m^3.

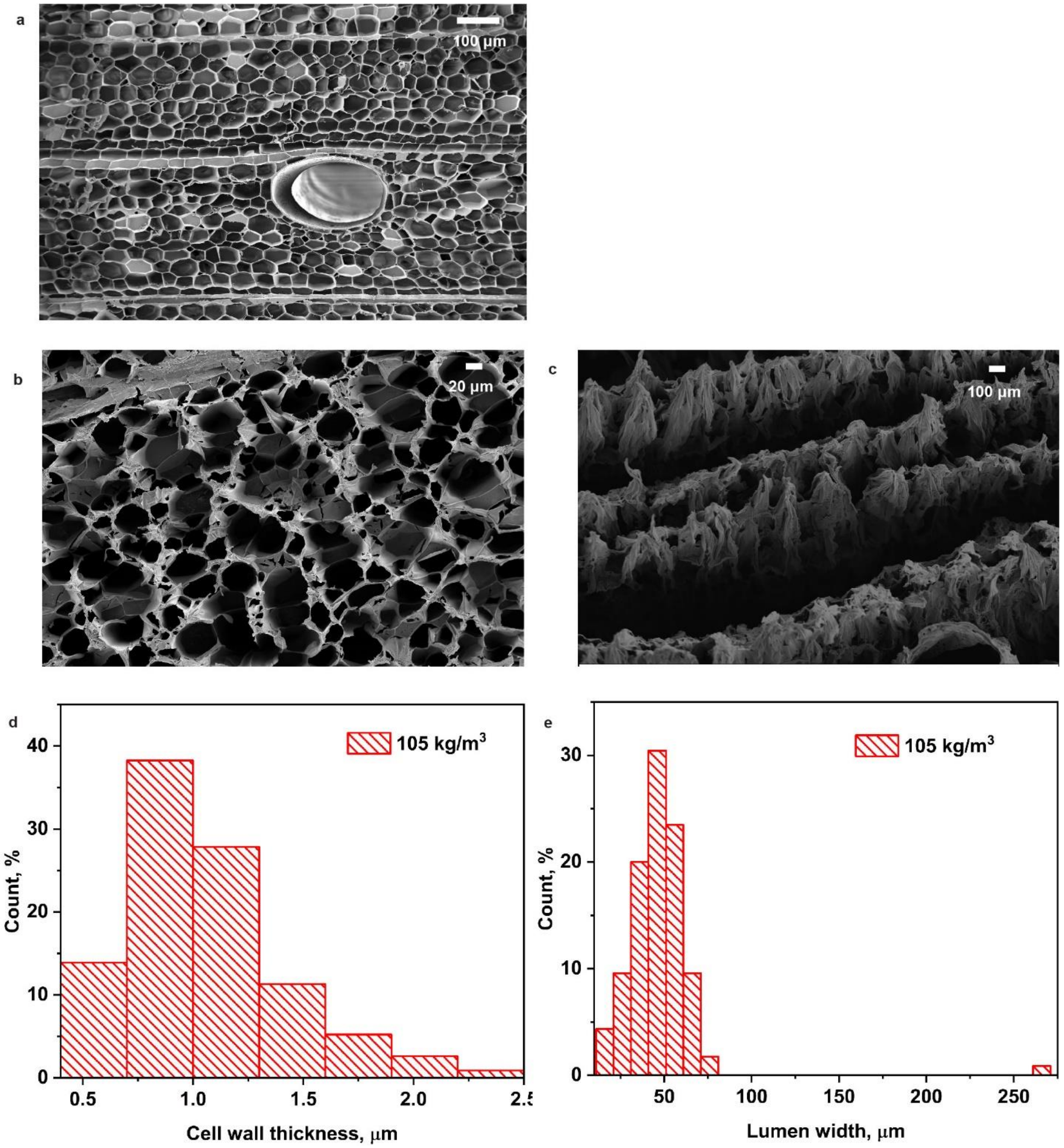

Supplementary Figure 16. Top-view SEM images showing the cross-section morphology of **a** wood (W), **b** cW and **c** cDW. Both cW and cDW were obtained at 1500 ºC. The distribution of **d** cell wall thickness and **e** lumen width in balsa wood (W) with a density of 105 kg/m^3.

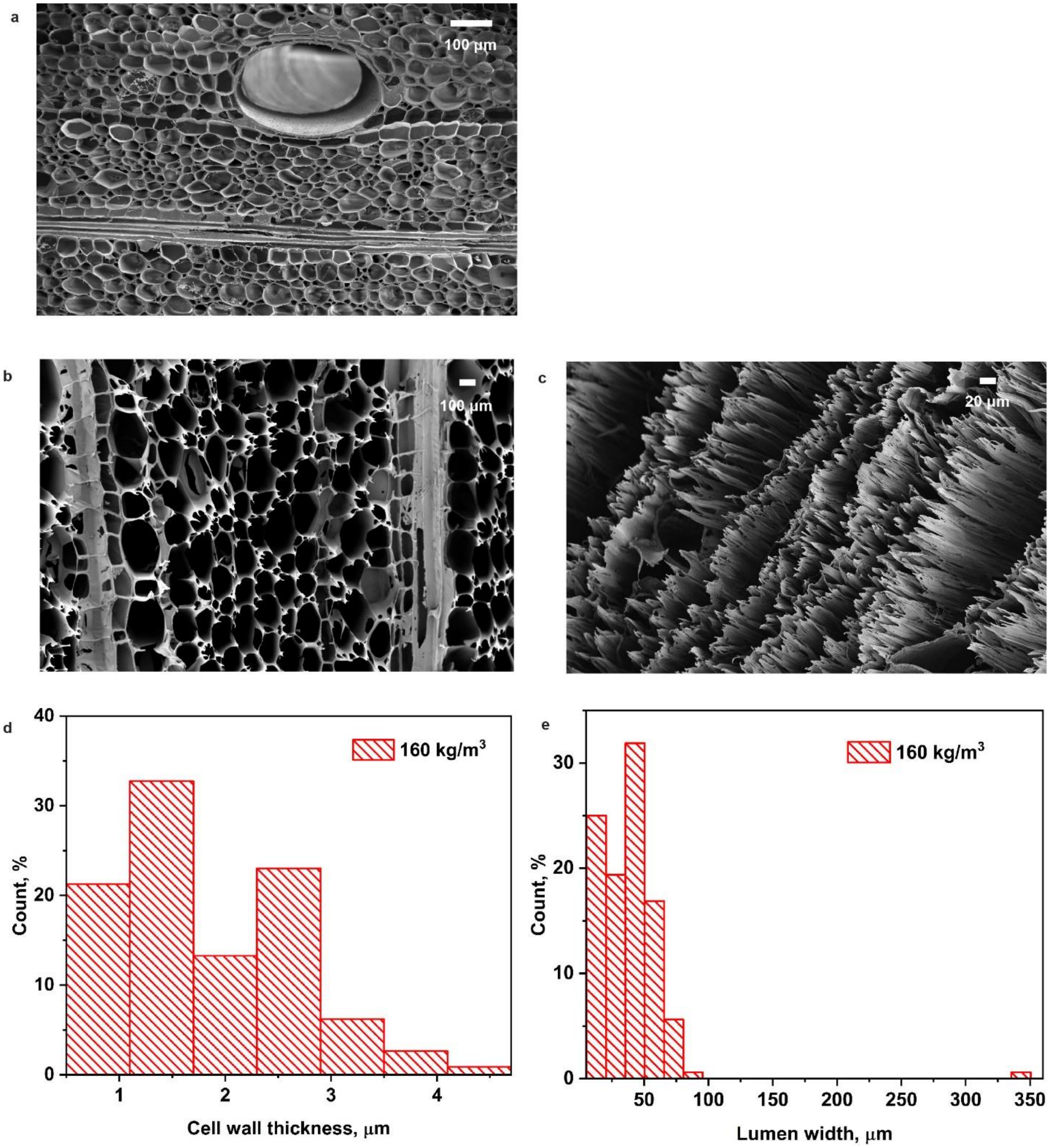

Supplementary Figure 17. Top-view SEM images showing the cross-section morphology of **a** wood (W), **b** cW and **c** cDW. Both cW and cDW were obtained at 1500 ºC. The distribution of **d** cell wall thickness and **e** lumen width in balsa wood (W) with a density of 160 kg/m^3.

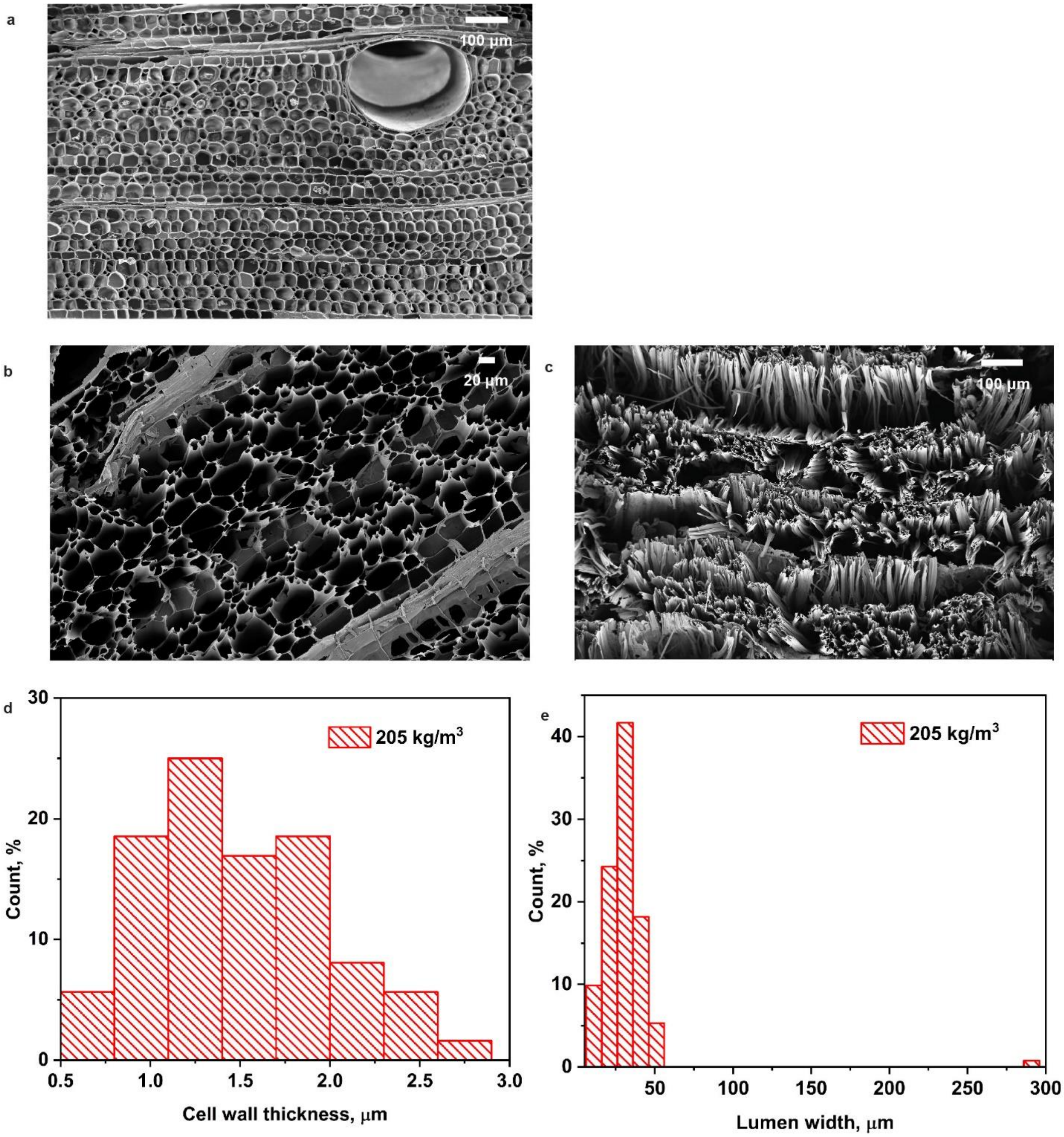

Supplementary Figure 18 Top-view SEM images showing the cross-section morphology of **a** wood (W), **b** cW and **c** cDW. Both cW and cDW were obtained at 1500 ºC. The distribution of **d** cell wall thickness and **e** lumen width in balsa wood (W) with a density of 205 kg/m^3.

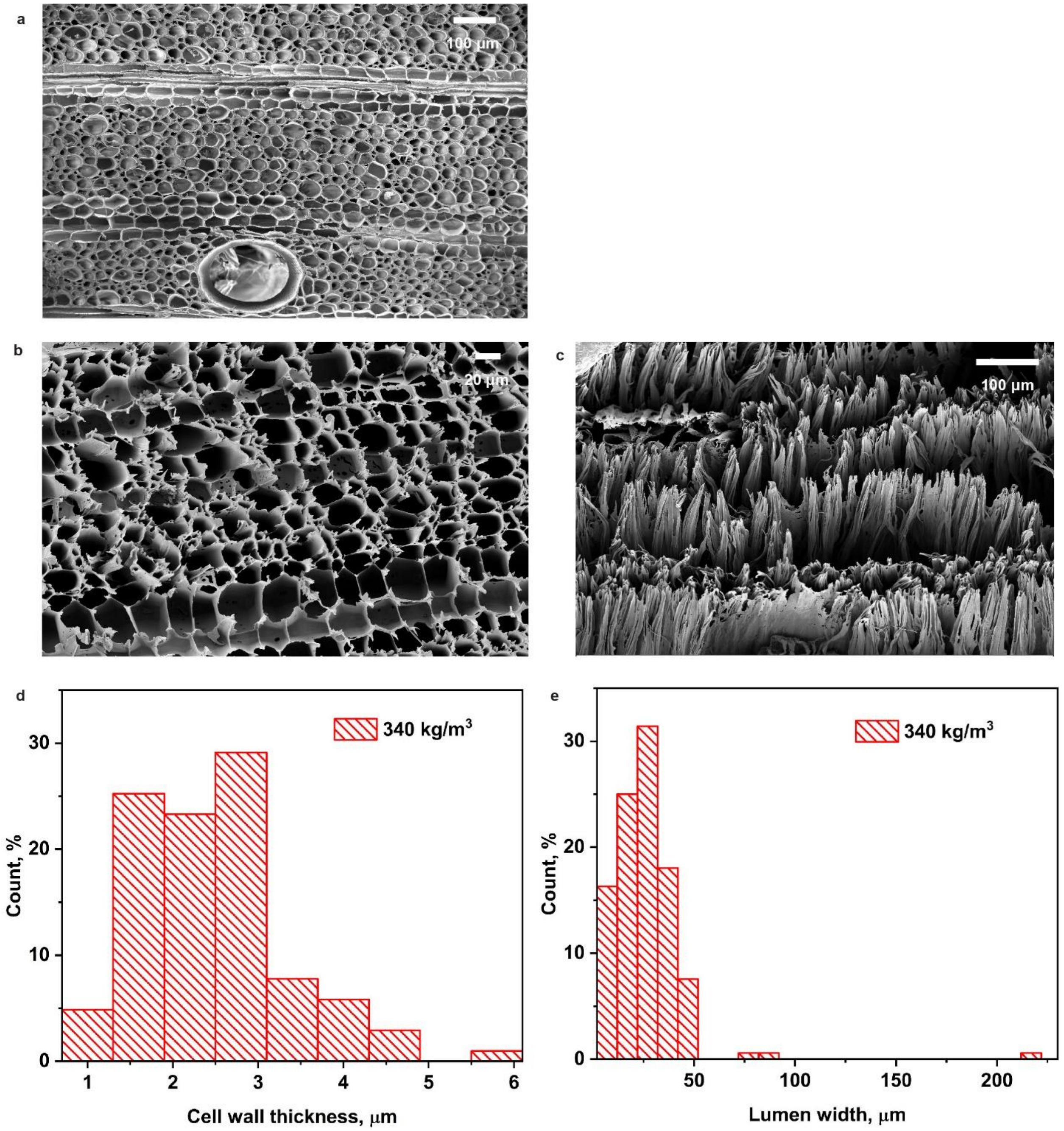

Supplementary Figure 19. Top-view SEM images showing the cross-section morphology of **a** wood (W), **b** cW and **c** cDW. Both cW and cDW were obtained at 1500 ºC. The distribution of **d** cell wall thickness and **e** lumen width in balsa wood (W) with a density of 340 kg/m^3.

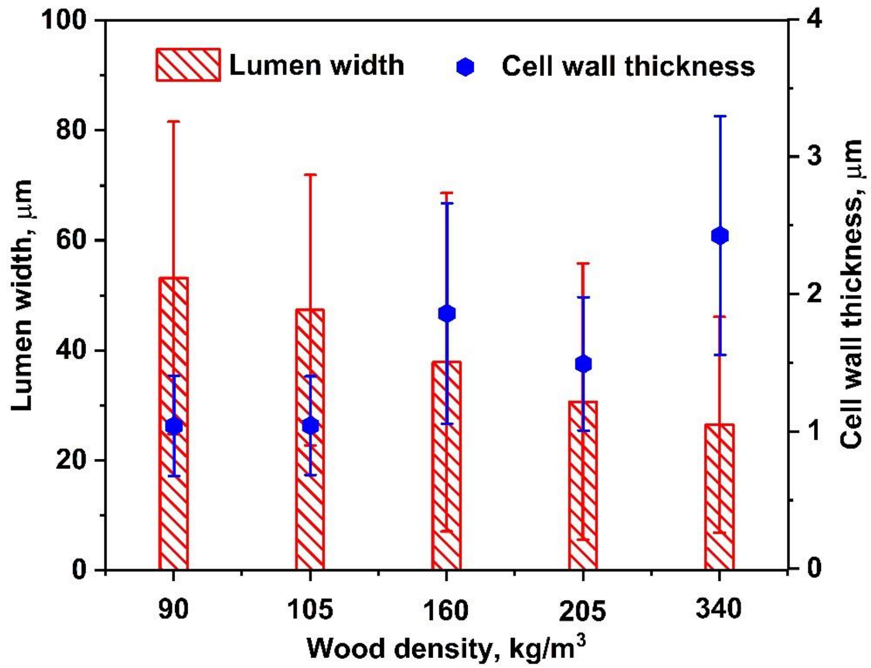

Supplementary Figure 20. The average lumen width and cell wall thickness of balsa wood with density spanning 90-340 kg/m^3. Error bars refer to standard deviation.

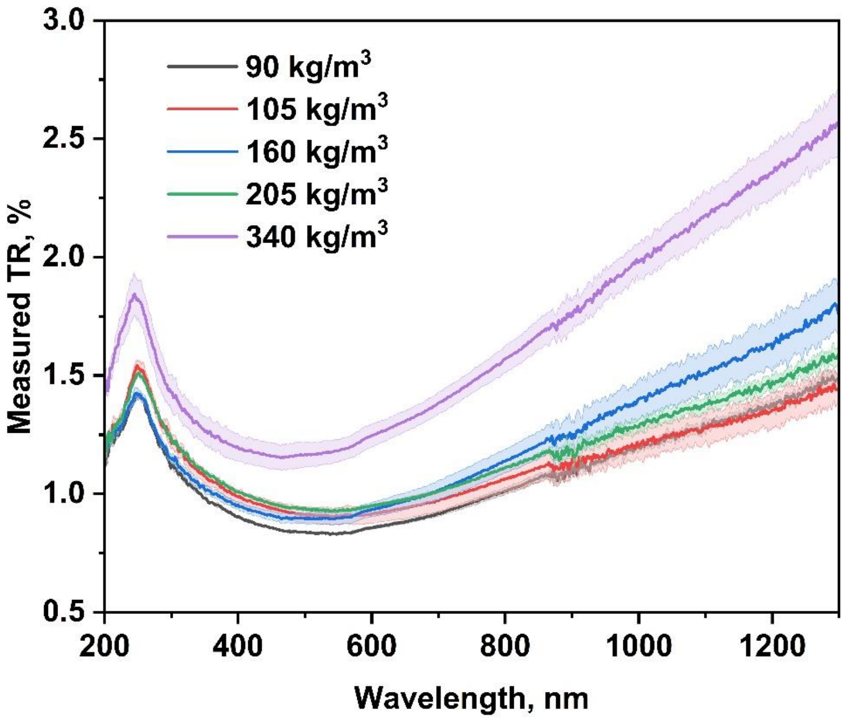

Supplementary Figure 21. Total light reflectance (TR) of cross section in cW obtained at 1500 ºC. The wood density spanning 90-340 kg/m^3. Shade colors represent standard deviation.

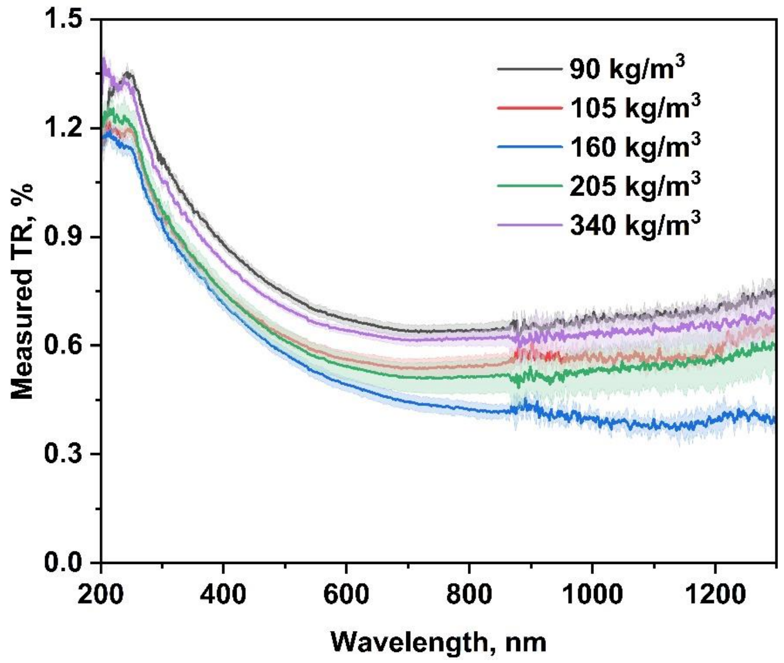

Supplementary Figure 22. Total light reflectance (TR) of cross section in cDW obtained at 1500 ºC. The wood density spanning 90-340 kg/m^3. Shade colors represent standard deviation.

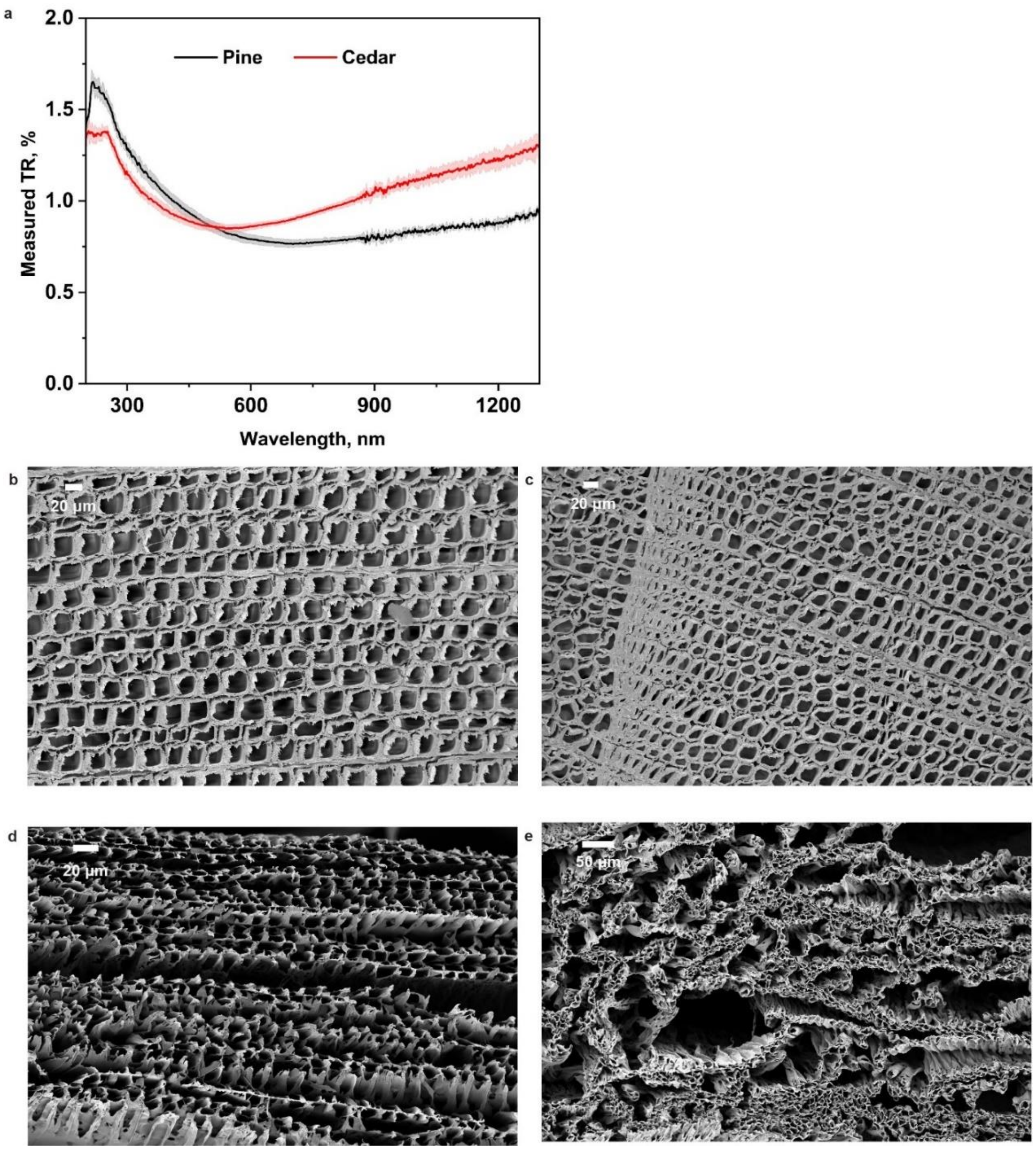

Supplementary Figure 23. a Total light reflectance (TR) of cross section in cDW obtained from pine and cedar wood. Top-view SEM images showing the cross-section morphology of **b** pine wood and **c** cedar wood, and cDW obtained from **d** pine and **e** cedar wood at carbonization temperature of 1500 ºC. Shade colors in **a** represent standard deviation.

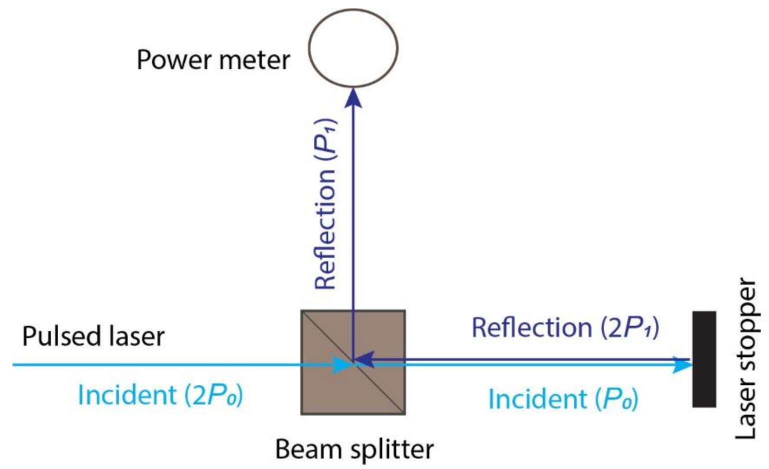

Supplementary Figure 24. A sketch of the setup used for reflectivity measurement of laser stopper.

Laser power (P_0) of both 50 mW and 100 mW (measured by power meter) illuminated laser stopper (Supplementary Figure 24) and power of reflected laser (P_1) are collected (Supplementary Table 1). The reflectivity (R_1) of the beam stopper was calculated by using Eq. 1.

$$R_1 = \frac{2P_1}{P_0} \times 100\ \% \qquad (1)$$

P_0 is the power of laser beam illuminating the laser stopper; $2P_1$ is the power of laser reflected by the laser stopper; P_1 is the power of laser that reached the power meter through the beam splitter; R_1 is the reflectivity of the laser stopper. The power (P_1) of laser reflection from the stopper was measured with five repetitions.

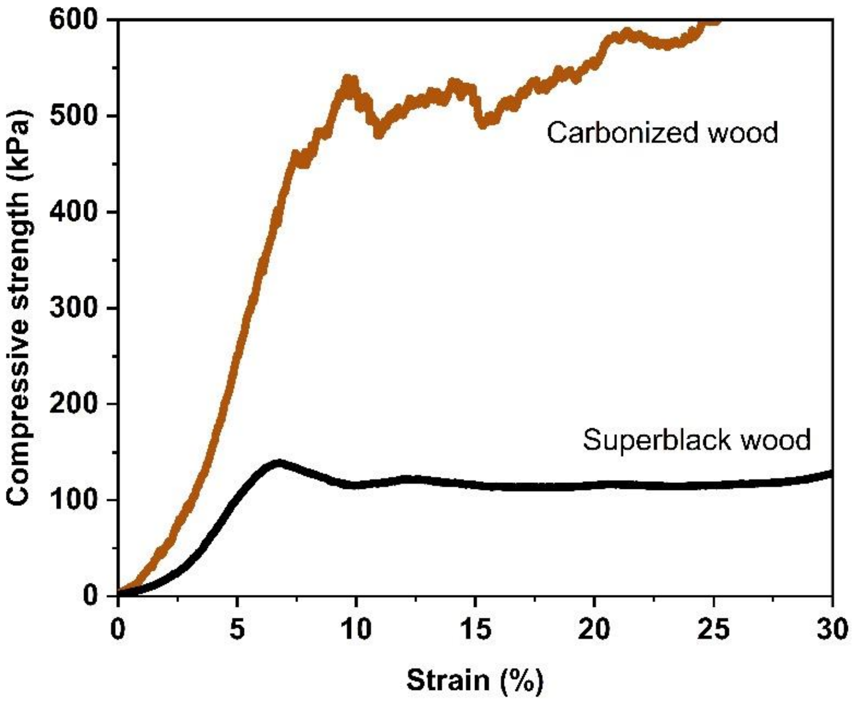

Supplementary Figure 25. Stress-strain curves of carbonized wood (cW) and superblack wood.

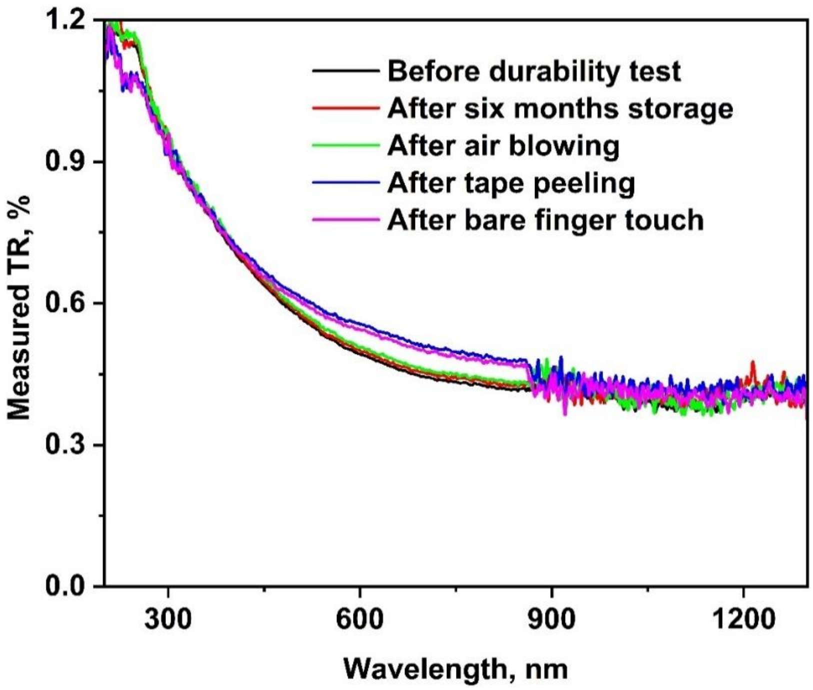

Supplementary Figure 26. Total light reflectance (TR) of cross section in superblack wood before and after six months storage, air blowing, tape peeling and bare finger touch test.

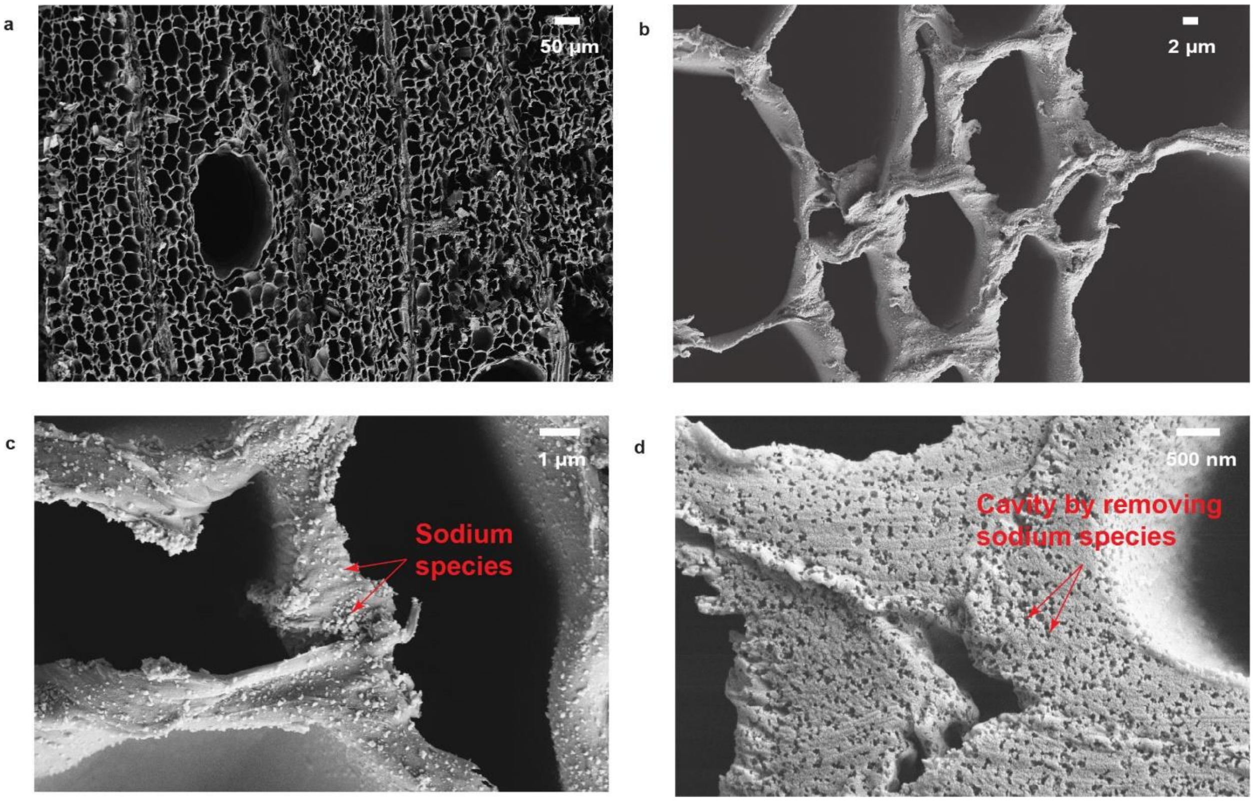

Supplementary Figure 27. SEM images showing the morphology of cross section in cDW obtained at 1500 ºC. The DW were prepared by using $NaClO_2$ solution and acetic acid with 6h delignification. **a-c** As prepared cDW. **d** cDW was further washed with 1 M HCl solution at 25 ºC overnight. The cavity was believed to be created by removing sodium species during the acid wash.

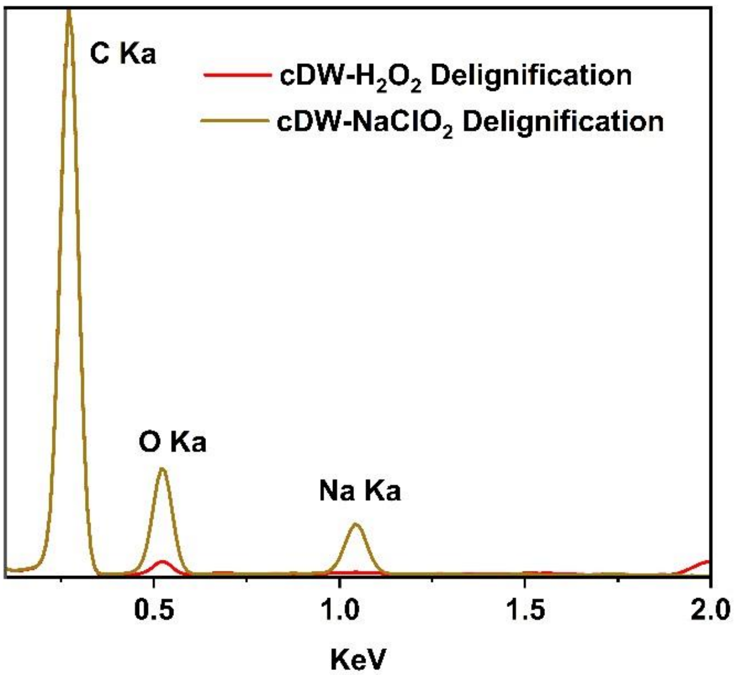

Supplementary Figure 28. Energy dispersive X-ray spectroscopy (EDS) spectra of cDW. The delignified wood (DW) was prepared using H_2O_2:CH_3COOH solution and $NaClO_2$ solution, respectively.

The EDS spectra clearly shows the presence of sodium species in cDW obtained from DW prepared from $NaClO_2$ solution, which is not the case for cDW obtained from DW that are prepared from metal-free delignification method (H_2O_2:CH_3COOH solution). The sodium species in $NaClO_2$ solution reside in the delignified wood matrix and account for 30 wt. % of mass fractions in the dried DW even after extended washing, which is measured gravimetrically. Note: in cDW (obtained from $NaClO_2$ delignification), high fraction of O species also come along with sodium species whose reduced form typically react with oxygen and water in air.

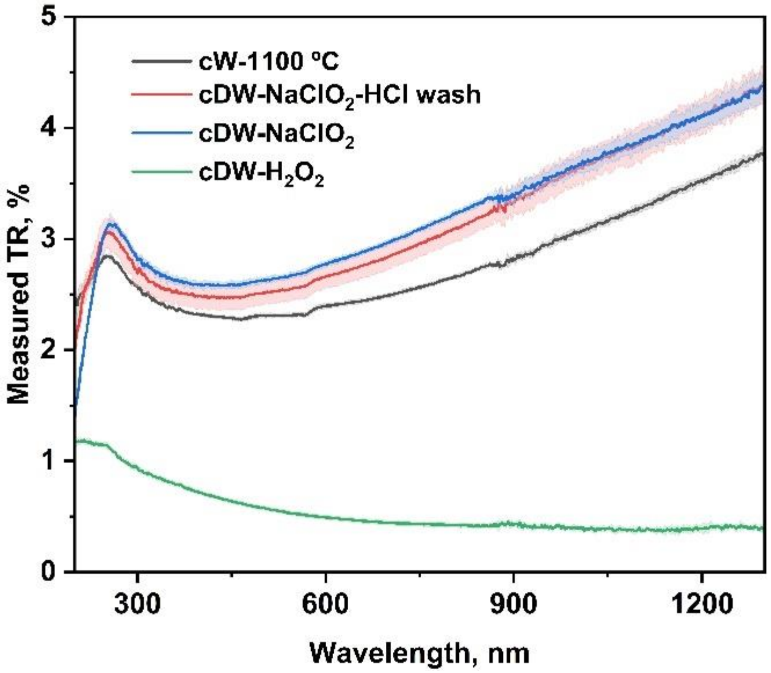

Supplementary Figure 29. Total light reflectance (TR) of cross section in cNW obtained at 1100 ºC (black line) and in cDW obtained at 1500 ºC (red, blue, and green lines). The delignified wood (DW) were prepared with different treatments: The DW were prepared by using $NaClO_2$ solution and acetic acid with 6h delignification (bule and red lines). cDW was further washed with 1 M HCl solution at 25 ºC overnight (red line). The DW were prepared by using H_2O_2 solution and acetic acid with 4h delignification (green line). Shade colors represent standard deviation.

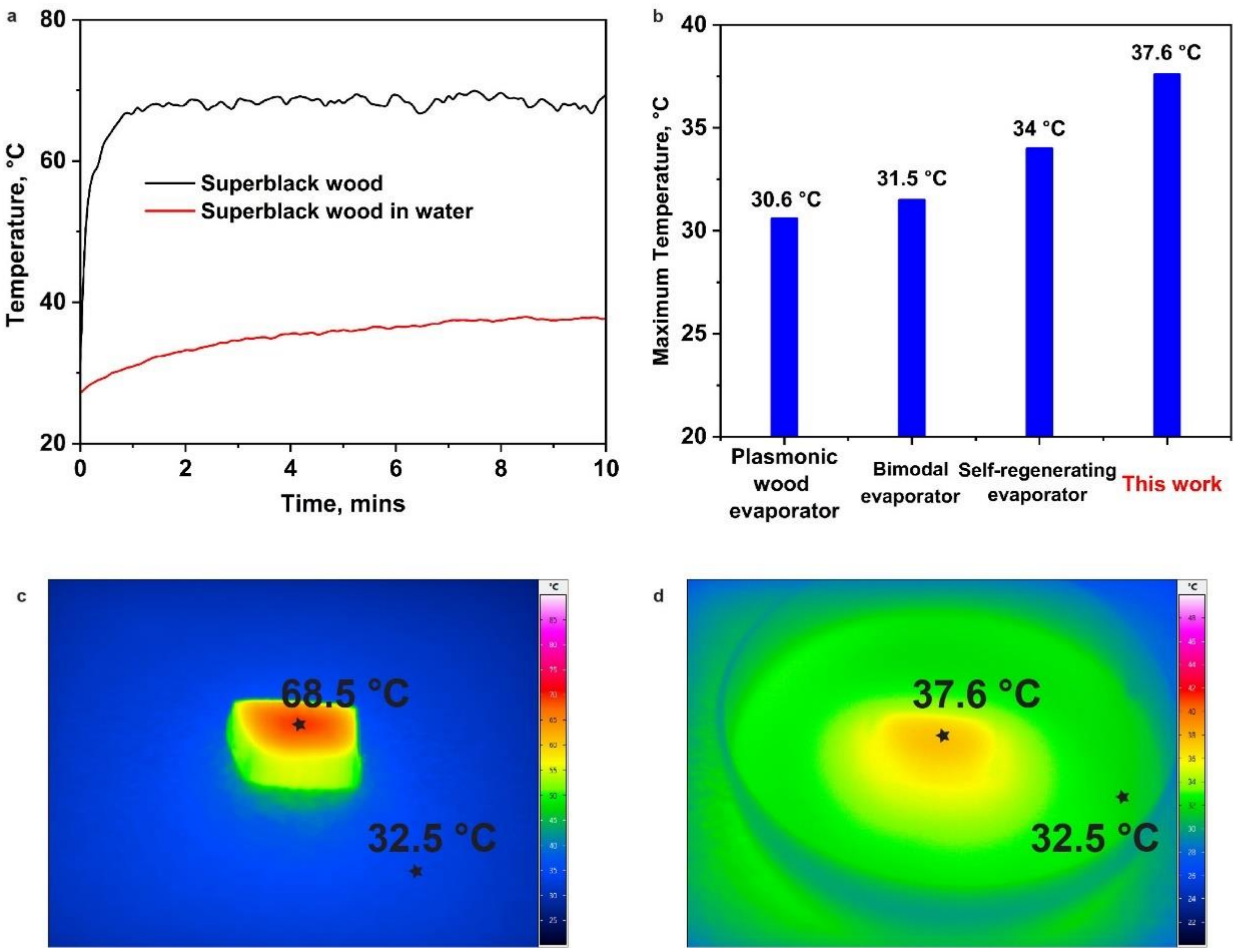

Supplementary Figure 30. a The temperature on the top surface of superblack wood and superblack wood in water under 1 sun of illumination (1 kW/m^2). **b** The stabilized temperature on the top surface of plasmonic wood evaporator[17], bimodal evaporator[18], self-regenerating evaporator[19] and superblack wood in water (this work); IR images of the **c** superblack wood and **d** superblack wood in water after 10 min under 1 sun illumination (1 kW/m^2).

Supplementary Section 3. Supplementary Tables and associated supplementary discussions.

Under the laser illumination (50 mW), very low scattered reflection (~16 ppm) was observed with superblack wood, which is lower than laser beam block (LB1/M) and 4 times lower than laser beam block (LB2/M). Under the strong laser illumination (100 mW), low scattered reflection (~30 ppm) was observed with superblack wood, which is lower than laser beam block (LB1/M) and 3 times lower than laser beam block (LB2/M) (Supplementary Table 1).

Supplementary Table 1. Measured reflection for laser stopper.

	Superblack wood		Laser block (LB2/M)		Laser block (LB1/M)	
P_0, mW	Reflected power (P_1), μW	Reflectivity (R_1), %	Reflected power (P_1), μW	Reflectivity (R_1), %	Reflected power (P_1), μW	Reflectivity (R_1), %
50	0.40 ± 0.01	0.0016	1.71 ± 0.04	0.00684	0.46 ± 0.01	0.0018
100	1.50 ± 0.03	0.0030	4.19 ± 0.12	0.00838	1.58 ± 0.03	0.0032

Supplementary References

1. *COMSOL 5.3: Wave Optics Module Model Application Manual*. https://doc.comsol.com/5.3/doc/com.comsol.help.woptics/WaveOpticsApplicationLibraryManual.pdf.
2. Taft EA, Philipp HR. Optical Properties of Graphite. *Physical Review* **138**, A197-A202 (1965).
3. *COMSOL Blog: How to Model the Optical Properties of Rough Surfaces* (2017).
4. Amemiya K, Koshikawa H, Imbe M, Yamaki T, Shitomi H. Perfect blackbody sheets from nano-precision microtextured elastomers for light and thermal radiation management. *Journal of Materials Chemistry C* **7**, 5418-5425 (2019).
5. Amemiya K, Shimizu Y, Koshikawa H, Shitomi H, Yamaki T. Supreme-black levels enabled by touchproof microcavity surface texture on anti-backscatter matrix. *Science Advances* **9**, eade4853 (2023).
6. Deinega A, Valuev I, Potapkin B, Lozovik Y. Minimizing light reflection from dielectric textured surfaces. *J Opt Soc Am A* **28**, 770-777 (2011).
7. Amemiya K*, et al.* Fabrication of hard-coated optical absorbers with microstructured surfaces using etched ion tracks: Toward broadband ultra-low reflectance. *Nuclear Instruments and Methods in Physics Research Section B* **356-357**, 154-159 (2015).
8. Borrega M, Ahvenainen P, Serimaa R, Gibson L. Composition and structure of balsa (Ochroma pyramidale) wood. *Wood Science and Technology* **49**, 403-420 (2015).
9. Borrega M, Gibson LJ. Mechanics of balsa (Ochroma pyramidale) wood. *Mechanics of Materials* **84**, 75-90 (2015).
10. Amemiya K, Fukuda D, Numata T, Tanabe M, Ichino Y. Comprehensive characterization of broadband ultralow reflectance of a porous nickel–phosphorus black surface by numerical simulation. *Appl Opt* **51**, 6917-6925 (2012).
11. Yang Z-P, Ci L, Bur J, Lin SH, Ajayan P. Experimental Observation of an Extremely Dark Material Made By a Low-Density Nanotube Array. *Nano letters* **8**, 446-451 (2008).
12. Wood BD, Dyer JS, Thurgood VA, Tomlin NA, Lehman JH, Shen T-C. Optical reflection and absorption of carbon nanotube forest films on substrates. *Journal of Applied Physics* **118**, 013106 (2015).

13. Lehman J, Yung C, Tomlin N, Conklin D, Stephens M. Carbon nanotube-based black coatings. *Applied Physics Reviews* **5**, 011103 (2018).

14. Tomlin NA, Curtin AE, White M, Lehman JH. Decrease in reflectance of vertically-aligned carbon nanotubes after oxygen plasma treatment. *Carbon* **74**, 329-332 (2014).

15. Yung CS, *et al.* Plasma modification of vertically aligned carbon nanotubes: Superhydrophobic surfaces with ultra-low reflectance. *Carbon* **127**, 195-201 (2018).

16. Brown RJC, Brewer PJ, Milton MJT. The physical and chemical properties of electroless nickel–phosphorus alloys and low reflectance nickel–phosphorus black surfaces. *Journal of Materials Chemistry* **12**, 2749-2754 (2002).

17. Zhu M, *et al.* Plasmonic Wood for High-Efficiency Solar Steam Generation. *Advanced Energy Materials* **8**, 1701028 (2018).

18. He S, *et al.* Nature-inspired salt resistant bimodal porous solar evaporator for efficient and stable water desalination. *Energy & Environmental Science* **12**, 1558-1567 (2019).

19. Kuang Y, *et al.* A High-Performance Self-Regenerating Solar Evaporator for Continuous Water Desalination. *Advanced Materials* **31**, 1900498 (2019).
